# Feature Selection for Chemical Sensor Arrays Using Mutual Information

**DOI:** 10.1371/journal.pone.0089840

**Published:** 2014-03-04

**Authors:** X. Rosalind Wang, Joseph T. Lizier, Thomas Nowotny, Amalia Z. Berna, Mikhail Prokopenko, Stephen C. Trowell

**Affiliations:** 1 CSIRO Computational Informatics, Epping, NSW, Australia; 2 CCNR, School of Engineering and Informatics, University of Sussex, Falmer, Brighton United Kingdom; 3 CSIRO Ecosystem Sciences and Food Futures Flagship, Canberra, ACT, Australia; University of California, Irvine, United States of America

## Abstract

We address the problem of feature selection for classifying a diverse set of chemicals using an array of metal oxide sensors. Our aim is to evaluate a filter approach to feature selection with reference to previous work, which used a wrapper approach on the same data set, and established best features and upper bounds on classification performance. We selected feature sets that exhibit the maximal mutual information with the identity of the chemicals. The selected features closely match those found to perform well in the previous study using a wrapper approach to conduct an exhaustive search of all permitted feature combinations. By comparing the classification performance of support vector machines (using features selected by mutual information) with the performance observed in the previous study, we found that while our approach does not always give the maximum possible classification performance, it always selects features that achieve classification performance approaching the optimum obtained by exhaustive search. We performed further classification using the selected feature set with some common classifiers and found that, for the selected features, Bayesian Networks gave the best performance. Finally, we compared the observed classification performances with the performance of classifiers using randomly selected features. We found that the selected features consistently outperformed randomly selected features for all tested classifiers. The mutual information filter approach is therefore a computationally efficient method for selecting near optimal features for chemical sensor arrays.

## Introduction

Feature selection is becoming increasingly important with the rapid increase in the speed and dimensionality of data acquisition in many fields. Typical application areas include for example sensor networks [1], [2], robotics [3], [4], astronomy [5] and bioinformatics [6]. Feature selection is the act of reducing the dimensionality of the data — be it from a sensor that collects a vast number of data points per sample, or data collected in parallel with many different sensors at the same time — to find the most useful subset of data for tasks such as classification, navigation or diagnostics.

In this paper, we focus on the problem of feature selection for classifying chemicals using an array of chemical sensors in an electronic nose. Our aim is to use an information-theoretic approach to feature selection (a filter method) on a data set with a large number of chemicals measured by an electronic nose. Previous work on feature selection with the same data set by Nowotny *et al.* [7] searched for the feature set with the best classification performance exhaustively (a wrapper method). We provide a direct comparison between the two approaches to feature selection and verify the classification performances of the selected feature sets. In other words, we evaluate the performance of our efficient filter method of feature selection using the upper limits provided by the exhaustive wrapper method search.

When selecting sensors for an electronic nose, the major considerations are the type and number of sensors to use, how to sample data from the chosen sensors and how to pre-process the collected raw data (see [8] for a recent review). Recent advances in solid-state chemistry using combinatorial synthetic approaches [9] or biosensor design based on natural genetic diversity [10], are now affording us an almost limitless repertoire of potential chemical sensors. It is therefore becoming increasingly important to identify optimal or at least sufficiently good subsets of potential sensors for incorporation into chemical sensor arrays (see [8], [11] for recent reviews).

An important aspect of the feature selection problem is that the optimal selection typically depends strongly on each particular application. It is therefore important to identify methods for feature selection that maximise the *value of the information obtained for reliable and acceptably-accurate classification*, rather than maximising information per se. The ultimate goal is to optimise classification accuracy at acceptable cost, which might be the price of sensors, communication costs, cost of processing and using the data, and any additional operation cost (e.g. [12]). The aspect of cost is, however, largely beyond the scope of this paper and will be considered in future work.

There are three general categories of feature selection methods: filter, wrapper, and embedded methods. In a filter method (e.g. [13]–[15]), features are selected based on some metrics that directly characterise those features and the process is independent of the learning and inference (eg. classification) algorithm. Consequently, feature selection needs only to be performed once, and different classifiers can be evaluated, making the filter method very appealing especially when the evaluation of the classification method is under consideration. In a wrapper method (e.g. [16], [17]), critical features are selected based on the success of the learning and inference algorithms. Consequently, the wrapper method often leads to better inferences as it bases its choice of features directly on the inference results. However, wrapper methods are in general much more time consuming (although it is common to adopt some greedy strategies, such as backward elimination or forward selection, to alleviate the computational cost [18]) than filter methods as they require executing learning and inference algorithms on all possible combinations of features. For example, the application of an SVM through a wrapper approach in [7] to the data set discussed here required 2–3 weeks of parallel computing time on a 70 processor modern supercomputer cluster, while the feature selection using the filter method took around an hour on a similar supercomputer cluster. Moreover, the previous work using the wrapper approach did not even include the use of separate validation and test sets, which would have further increased the necessary computation time in crossvalidation. This lack of a separate validation set adds to the risk of over-fitting in the wrapper based results.

A third feature selection method is the embedded approach, where the feature selection is built into the training process of the classifier (e.g. [19], [20]). Thus, the embedded approach is similar to wrapper method in that it is specific to a given learning algorithm. However, the embedded approach is more computationally efficient as it makes better use of available data and avoids retraining from scratch for every possible combination of the feature set [18].

In this paper, we aim to evaluate the effectiveness of a filter approach to feature selection, due to its advantage of reducing computational cost and independence of the classifier, in comparison to previous work using a wrapper approach. In the field of olfactory sensors, various metrics based on signal processing have been proposed for feature selection via filter methods: Raman *et al.* [21] selected the subset using a measure derived from Fisher's Linear Discriminant Analysis, Muezzinoglu *et al.* [22] explored the use of the squared Mahalanobis distance, and Vergara *et al.* [13] computed the frequency responses of the signals by first calculating the input-output cross-correlations and then calculating the Fourier Transform of the responses. Feature selection based on machine learning and artificial intelligence techniques has also been explored by various groups. Among these techniques, Genetic Algorithms (GAs) are popular (e.g. [15], [23], [24]) due to the approach's resilience to becoming trapped in local optima. While the studies above showed good classification results from the feature selection, in most cases only one or two classifiers were tested. Furthermore none of the studies compared the results with an exhaustive wrapper-based search of features.

Another approach to filter-based feature selection relies on information theory. First derived by Shannon [25], information theory provides a measure of the uncertainty associated with the data, allowing us to quantify what is meant by “information collected by the sensors”. Consequently, it is a natural measure to be employed in selecting the best features independently from the classification methods to be used. Methods based on information theory have been used quite widely to optimise, for example, optimal geospatial arrangement of sensor arrays. Krause and Guestrin [26] introduced a criterion which minimises the conditional entropy of all the possible sensors given the subset chosen. These authors also proposed a criterion maximising the mutual information between the subset and the rest of the sensors [14]. Olsson *et al.* [1] suggested a method that uses a combination of mutual information and information metric to describe an information coverage (IC), which balances redundancy and novelty in the data. More recently, Wang *et al.* [27] explored the selection of sensors by maximising the entropy in the subsets and showed, using a simple data set, that the different sensor selection metrics selected very similar subsets. In all the cases above, the objective was to select the optimal spatial placement of sensors such that the maximum amount of data is gathered, the optimisations were not done for the purpose of classification of some target variable.

In the field of artificial olfaction, the use of information theory for optimising a sensor array first appeared a decade ago in a paper published by Pearce and Sánchez-Montañés [28]. In this paper, the authors showed that Fisher Information can be used as a lower bound for the classification performance of the sensor responses. Vergara *et al.* [29] optimised the operating temperature for a single sensor by maximising the Kullback-Leibler distance (KL-distance) with respect to a single parameter, the sensor heater voltage, for a given set of odours. The paper compared the results with that of Mahalanobis distance (MD) and found that the optimal parameter identified by the KL-distance led to better classification performance in the considered six-class problem.

Our aim was to evaluate the performance of *mutual information* (MI), as an efficient and conceptually relevant filter approach, in feature selection for a chemical sensor array across a range of classifiers and in relation to a computationally exhaustive wrapper approach applied to the same data set in the recent study by Nowotny *et al.* [7]. Mutual information is a measure of the amount of information one random variable contains about another [30]. The MI between two variables tells us the reduction in uncertainty of one due to the knowledge of the other. In the case of feature selection, maximising the MI among variables gives us the subset with the most *novel and non-redundant* information about the sensor data (see Methods for full details of the criterion).

The use of mutual information for feature selection in classification problems was first suggested by Battiti [31]. Recently, Fonollosa *et al.* [32] used Mutual Information (MI) to optimise the operating temperature of four metal-oxide sensors when measuring four different gases at various concentrations. To calculate the MI, the authors quantised the continuous responses of the sensors. The authors showed that sensor arrays with different numbers of sensors have different maximum MI over a range of gas concentrations. The paper concluded that the MI approach can be used to quantify the classification abilities of the sensors, however, no classification results were shown to verify this claim.

It is known that MI from a large set of features with a small amount of data can be difficult to estimate, and also there can be a large number of potential feature sets to evaluate [31], [33]. Peng *et al.* [34] attempted to address these issues by computing minimal-Redundancy-Maximum-Relevance (mRMR) of candidate features with the class values. The mRMR algorithm does not consider all possible features in every size constraint, rather, it iteratively selects new features by minimising their pairwise redundancy with features chosen in previous steps and maximises their relevance to the class, thus reducing the computational cost. The authors showed that their approach efficiently selects good feature sets for multiple classification methods in 4 different data sets. Similarly to the mRMR algorithm, Rodriguez-Lujan *et al.* [35] formulated the problem of solving minimum redundancy and maximum relevance for the feature selection of large data sets using a method named Quadratic Programming Feature Selection (QPFS). The authors introduced an objective function with quadratic and linear terms to capture the pairwise dependence between the variables and novelty between the feature and class. The QPFS algorithm solves the quadratic programming problem using the Nyström method to improve computational speed. The authors showed that the algorithm can achieve similar accuracy to previous methods but with higher efficiency. Recently, Pashami *et al.* [2] modified the QPFS algorithm by applying the Fisher index, a ratio between the mean and standard deviation of the samples, to compute the relevance of the data. The authors applied the modified algorithm to data from an array of metal oxide gas sensors, similar to those used in this paper, for the detection of changes in distant gas sources.

In this paper, we will evaluate MI exhaustively for all potential feature sets despite the aforementioned issues with its efficient estimation and with potential computational cost in general. In contrast to directly estimating MI from the candidate set of features, the pairwise approaches [34], [35] discussed earlier use only heuristics for minimising redundancy and do not capture synergies of the features with the class (See Methods for further details). Addressing redundancy and synergy simultaneously is key in designing devices with the minimum number of sensors with the best possible classification performance, and can only be achieved with a full multivariate evaluation. Also, we use a recently developed nearest-neighbours technique for estimating MI [36], which has been demonstrated to be efficient for a relatively large number of variables. This technique allows us to utilise the full range of responses of the sensors, which is advantageous over the quantisation process employed by Fonollosa *et al.* [32]. Finally, we have a relatively small number of features, thus the evaluation of MI for all possible combinations of features is computationally tractable, aligning with our goal of comparing feature selection via our best possible MI estimates with the wrapper approach that was based directly on classification.

Without a direct comparison with mRMR and QPFS we do not claim that these less computationally intensive algorithms would significantly under-perform for a given dataset. Therefore, a choice between (a) a more comprehensive information theoretic approach advocated in this paper and (b) a simpler pair-wise approach that alleviates the computational cost of the algorithm remains an open question. Typically, such a question would need to be resolved when a particular trade-off between computational efficiency and classification accuracy can be established for specific datasets.

Feature selection and subsequent classification was performed on data from 12 metal oxide sensors used to make 10 replicate measurements of 20 chemical analytes, each at a fixed concentration. The features to be selected comprise the 12 sensors and 6 candidate time points taken from the 2 Hz data stream generated during sample presentation. Each combination of an individual sensor with a candidate time point can be viewed as a virtual sensor or “feature”. This data set is the same as used by Nowotny *et al.* [7], who employed a wrapper method to exhaustively search through all permitted combinations of sensors and time points to find the best feature sets. Here, we employ a maximum MI criterion to find feature subsets. We then compare the resulting feature sets, as well as their classification performance, with those reported from the exhaustive search in [7]. Finally, we also perform classification with the selected feature sets using a variety of common classifiers in addition to the linear support vector machines used in [7] and compare the results across classifiers.

## Results

We evaluate our feature selection method in 10 fold cross validation. The data set of 200 measurements is randomly split (many times) into a training set of 180 measurements and a test set of the remaining 20 measurements. For each such split into a training and test set, we first identify an optimal feature set using the training data. These features are then extracted from the full measurements of both the training and test sets. We then test numerous different classifiers by training them on the resulting training data and evaluating them with the test data. The observed performance of the entire feature selection and classification process, as well as a comparison to the classification performance in the exhaustive search in [7], are reported below.

### Optimal Feature Selection

**Figure 1:**
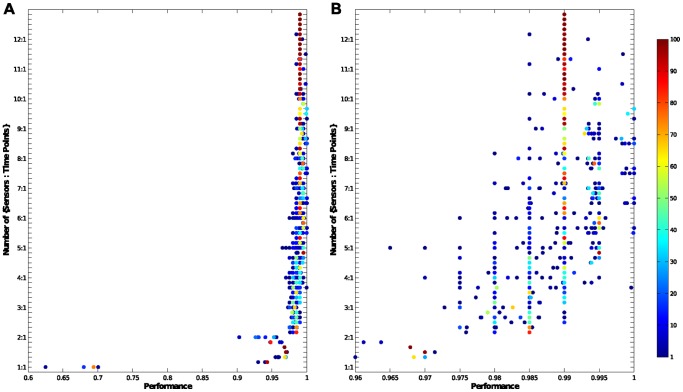
Classification performance from [7] using the feature subset identified by MI. (a) All the results; (b) Zoomed in to classification performance of between 0.96 and 1. The y-axis shows the number of {sensors∶time points} chosen. For example, 4∶1 represents finding the optimal subset of 4 sensors and 1 time point in each sensor. The tick marks in between the labels on the y-axis represents the other number of time points options (e.g. the tick marks between 4∶1 and 5∶1 are {4∶2, 4∶3, 4∶4, 4∶5, 4∶6} respectively. The position on the x-axis indicates the performance of an optimal subset of features. Each dot on the plot represents a specific feature subset, positioned according to its classification performance. The colour of the dots represents the percentage of occasions when that optimal subset of features was chosen (via the MI measure). Note that if two optimal subsets had the same classification results, then their numbers were combined and combined result was shown on this plot.

We first looked at the optimal feature set as defined by the maximum MI criterion for all possible “size constraints”, i.e. a given number of sensors and time points per sensor (where the selected time point(s) are held in common for all sensors in a given set). Feature selection was performed for each training data set separately. We observed that, for most size constraints, several different optimal feature sets were discovered for different splits of training and test set. While there are optimal feature sets which were only discovered for a single training data set, for almost all size constraints, over 90% of the training data sets lead to one of three or fewer most prevalent optimal feature sets (see Figure 1). Both observations are likely related to the particular properties of our data set. Since there are many redundancies in the sensor readings (10 measurements of each chemical sample), it is not surprising that many of the training sets will give the same feature selection as they will contain roughly the same type of measurements. At the same time, we have a fairly small data set (200 samples), meaning that small changes in the inclusion of one or two measurements may cause a large enough change in the overall mutual information value to alter the selection of certain features, thus giving us several distinct optimised feature subsets.

**Figure 2:**
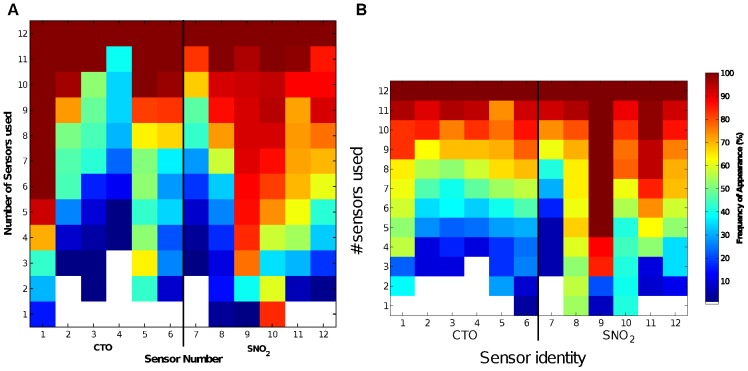
Frequency of appearance of individual sensors in selected feature sets. (a) Sensors selected by the mutual information criterion discussed in this paper, (b) sensors in the “top 10” group of best classification performance through exhaustive search (from [7], reproduced here for reader convenience). The vertical bar between sensors 6 & 7 separates CTO and SnO_2_ sensors. The y-axis marks the number of sensors used, and the x-axis the identity of the used sensor, where sensors 1–6 were CTO sensors and 7–12 were SnO_2_. The colour shows the relative frequency (%) with which the particular sensor appeared in feature groups of the corresponding size, e.g. dark red at (x = 1,y = 6) in (a) indicates that sensor 1 was used 100% of the time in feature selections with 6 sensors each.

We then asked: which sensors and which features (sensor-time point combinations) were particularly useful for our classification task and how did this depend on the number of features used overall? Figure 2A shows the frequency with which each sensor (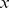-axis) appeared in an optimal feature set for each sensor size constraint (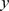-axis). From this figure, we notice several prominent aspects of the optimal feature sets chosen.

Firstly, while all the sensors were present in at least one optimal set for size constraints of more than three sensors, some were selected much more frequently than others. In particular, sensor 1 and sensor 9 occur more often than not in all size constraints of 4 or more sensors. It is interesting to note that these two sensors represent one each of the two different types of sensor surface chemistry used (cf. Table 1 in [7]). This shows that the two different sensor technologies offer unique and non-redundant information with respect to each other, providing novelty when the chemical classes are considered in the mutual information calculation. Similarly, some sensors were selected much less often than the others. In particular, sensors 4 and 7 were under-represented. They too, represent one each of the two different sensor technologies used in the experiment.

**Table 1:** Analysis of the balance between sensors of different chemistry used in selected feature sets.

No. Sensors	% CTO only	% SnO_2_ only	% CTO>SnO_2_	% SnO_2_>CTO	% CTO = SnO_2_
1	14.3	85.7	0.0	0.0	0.0
2	0.0	19.0	0.0	0.0	81.0
3	0.0	2.2	33.7	64.2	0.0
4	0.0	2.0	11.0	52.7	34.3
5	0.0	0.0	17.7	82.3	0.0
6	0.0	0.0	9.0	43.0	48.0
7	0.0	0.0	45.3	54.7	0.0
8	0.0	0.0	35.3	51.5	13.2
9	0.0	0.0	42.2	57.8	0.0
10	0.0	0.0	31.3	52.0	16.7
11	0.0	0.0	38.2	61.8	0.0
12	0.0	0.0	0.0	0.0	100.0

Each row shows the composition of the feature sets selected by MI given the sensor size constraint, the columns give the percentage of the total selected feature sets with the labelled sensor contents.

Secondly, in general as the size constraints were relaxed, the number of times a sensor was used also increased. This may be at least partly explained by the observation in [27] (using maximum entropy for feature selection), that as the size constraints relax, more new sensors are added to previously-selected sets of smaller size constraints (rather than a whole different set of sensors being selected at the larger size). This situation would be advantageous when applying our sensor selection criteria to practical situations: for example, if a sensor array for detecting a particular disease was to be manufactured at different costs, then the versions for each cost of production (i.e. optimised for a different number of sensors) could be efficiently manufactured by incremental addition of sensors from within the same (reduced) set of sensors. This observation can also help with future feature selection problems when the number of features is so large that evaluating MI for all possible combinations of features becomes intractable: in such cases it would be possible to iteratively select new features as the size constraint increases, knowing that the full feature set for the new size constraint would likely be the optimal or near-optimal set if all combinations were evaluated.

**Figure 3:**
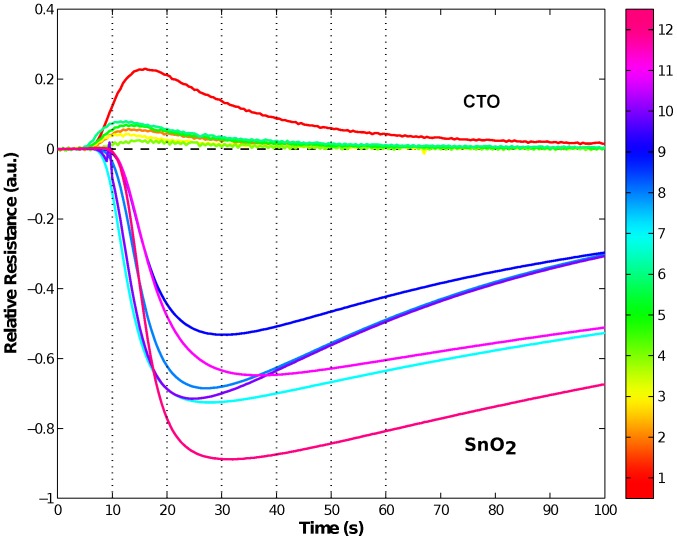
Example FOX Enose responses from the twelve-sensor array. Colour bar on the right shows the sensor number, where 1–6 are CTO sensors and 7–12 are SnO_2_ sensors. Responses of SnO_2_ sensors are drawn downwards and responses of CTO sensors upwards. The vertical dashed lines mark the time points where the data for the study was extracted. The sensor readings here show the response to 1-pentanol. Note that only the first 100 seconds of the full 300 seconds of data are shown here.

Lastly, we notice that the SnO_2_ sensors are included in the optimal feature sets more often than the CTO sensors across almost all size constraints. This is shown in Table 1 where we calculated the percentage of total optimal feature sets containing various sensor compositions. From this table, we can see that (for a given number of sensors) there are always more selected sets where the SnO_2_ sensors outnumber the CTO sensors. One possible reason for the prevalence of the SnO_2_ sensors could be merely because they are more sensitive than the CTO sensors (see Figure 3). However, sensitivity is only one aspect of sensor quality and may be less important than sensor noise and specificity, when it comes to analyte classification.

Another interesting observation in Table 1 is that, except for sensor size constraints with very few sensors, there are no feature sets where only one type of sensor technology was found in the optimal feature sets. This is consistent with the observation that the pair of most and the pair of least frequently used sensors each contained representatives from both classes of sensor chemistry. This strengthens the evidence that the two sensor chemistries provide unique and non-redundant information with respect to each other.

**Figure 4:**
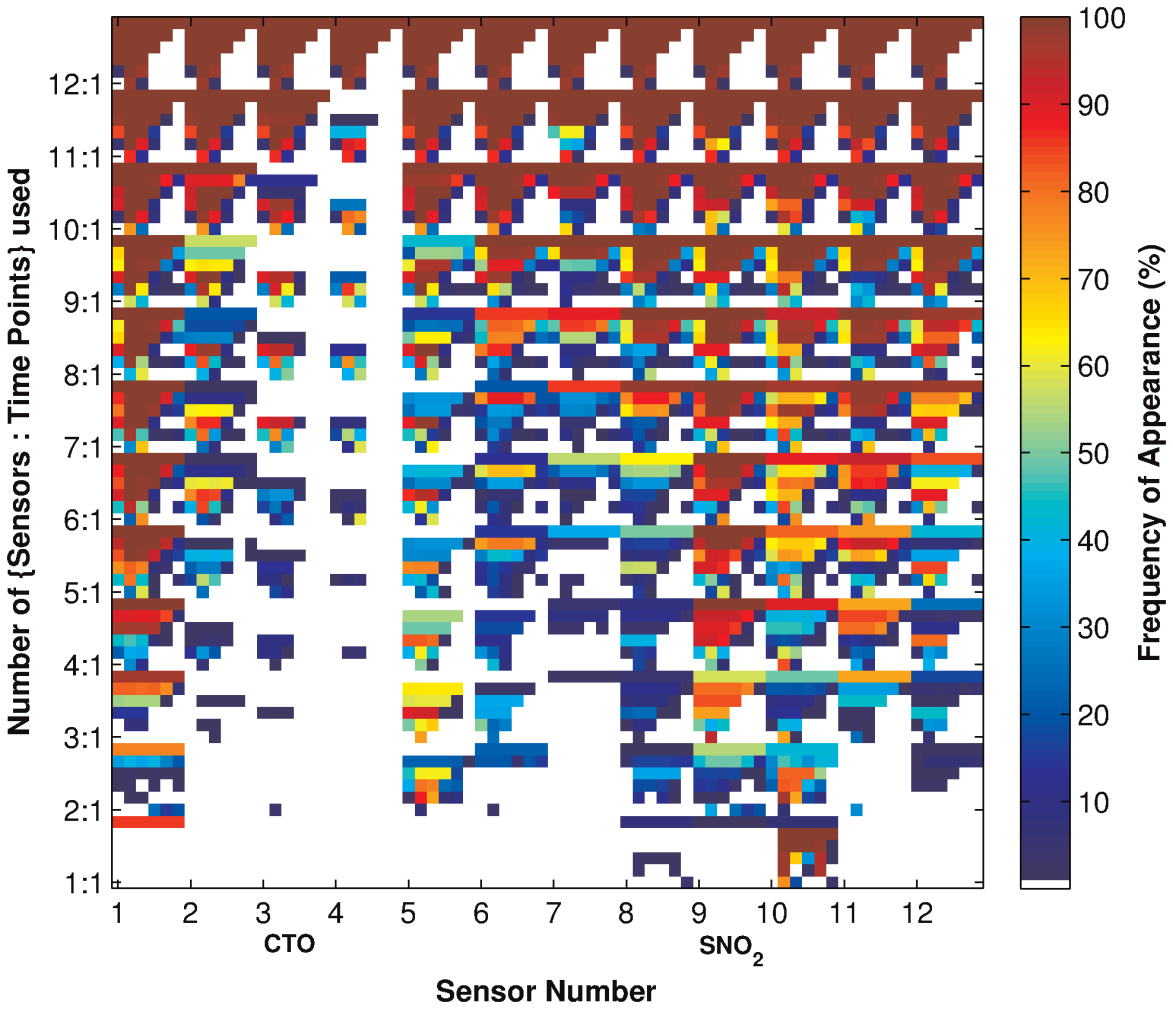
Frequency of appearance of individual features in selected feature sets. The y-axis indicates the number of {sensors∶time points} used. For example, 4∶1 represents finding optimal subset of 4 sensors and 1 time point in each sensor. The rows in between the labels on the y-axis represents the other options for the number of time points (e.g. the rows between 4∶1 and 5∶1 are {4∶2, 4∶3, 4∶4, 4∶5, 4∶6} respectively. The x-axis marks the identities of the sensors and time points that were used. Colours represent the frequency (%) with which a {sensor∶time point} combination was chosen, as shown in the colour scale.

Figure 4 shows each feature's frequency of appearance given the size constraints in both the number of sensors and time points. This figure gives a finer understanding of the features selected. Again, we notice several aspects: Firstly, for time point constraints of 1, over almost all sensor size constraints, time point 2 or 3 (i.e., the data gathered at 20 and 30 seconds) is chosen for the optimal feature sets. From Figure 3, we can see that these are times just before the maximal responses of the sensors, where we should expect the maximum amount of variation in the data giving us the largest values in MI.

The second point of interest was noticed earlier in the sensor only constraints, that is, in most cases, as the size of time point constraints increases, more features were added to existing sets. The exceptions are sensors 3 and 4, which do not get chosen for time point constraints of more than 4 for many of the sensor number constraints. This can be explained by looking closely at the data from these sensors for 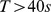, as the readings from these sensor hovers just above 0 from this time point onwards. This means while at small 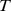 these two sensors provide information on the chemicals, they do not provide any information for large 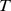, thus they are included in those constraints with a small number of time points but not for those with a large number.

### Comparison with the Wrapper Method of Feature Selection

The optimal subsets of features found using our MI criterion were compared with the results reported in [7]. In both methods, the SVM cost parameter of 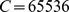 is used. The authors of [7] compared classification results of 4 different 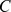 values and found consistent performances, thus report only the performance of the largest 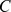 value. We tested the performance of the linear SVM with 9 different 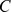 values (see Methods: Classification Methods), and found that 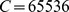 performs better than or consistently with the other 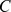 values (see Figure S1). Therefore, we compare the two feature selection methods using the same 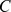 value.

**Figure 5:**
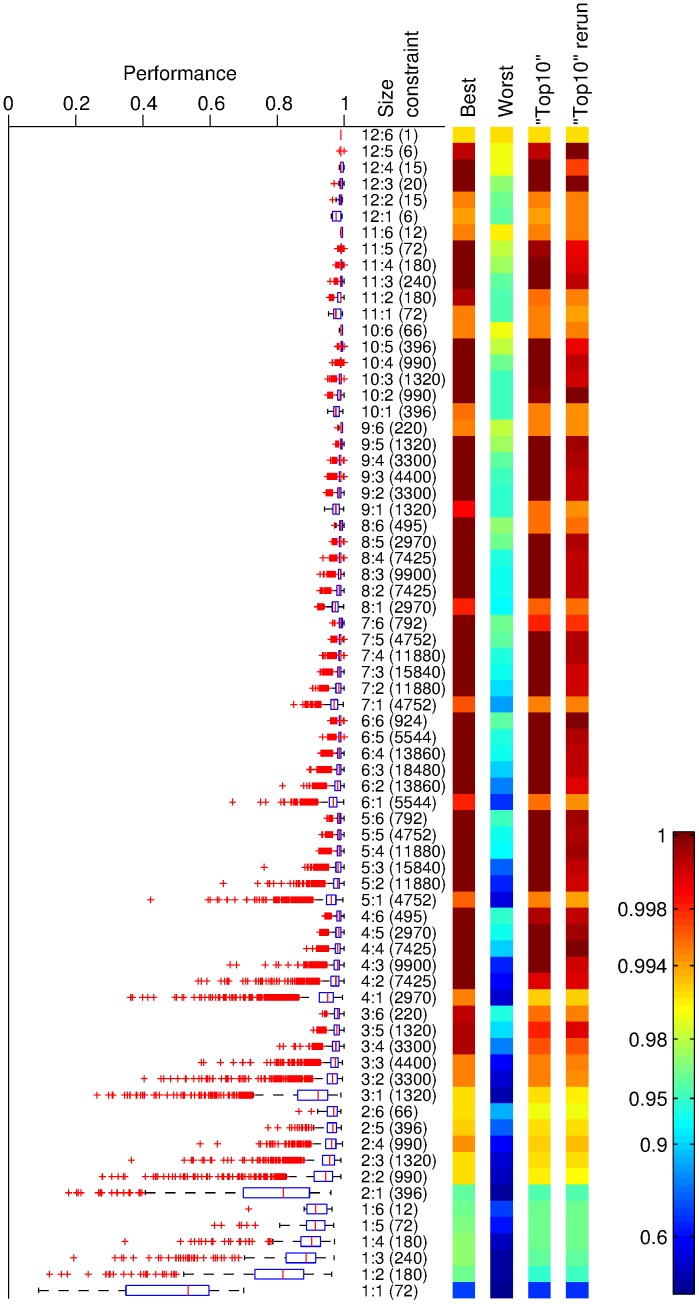
Fractional prediction accuracy of 10-fold cross-validation using linear SVMs for all allowed combinations of 12 sensors and 6 time points. The box plots show the median, 25% and 75% quantiles, the estimated overall range (whiskers) and identified outliers (red crosses) of all the combinations (the total number of which is shown in the ‘constraint’ column) of feature sets. The size constraints are shown on the right in order of number of sensors and time points. The coloured columns show best and worst classification performances of all the feature sets, as well as the averaged performance of the “top 10” group, and repeated 10-fold cross-validation results of this “top 10” group. Results from [31] reproduced here for reader convenience.

While both methods use 10 repetitions of ten-fold balanced cross-validation for measuring classification performance, there is one subtle difference in the execution: In our method here, for each of the 100 training sets, we calculate the optimal feature set using the MI criterion for that training set of 180 data samples and then use the identified feature set to determine the classification performance for the corresponding test set of 20 data samples. We eventually report the average of all observed performances. In the wrapper approach in [7], classification performance is calculated across all 100 training and test set splits for each possible combination of feature sets of a given size constraint. The 10 feature sets with the best so determined classification performances are selected as the optimal feature sets, or the “top 10” group and their average achieved classification performance is reported. Note that the reported performances do not include the use of separate evalutation and test sets and may, therefore, not accurately reflect the expected performance on entirely new, unseen data, due to the known risks of overfitting. We display the relevant data from [7] in Figures 2B and 5 for easy reference and comparison.

First, we compare the sets selected by maximum MI criterion (Figure 2A) with the “top ten” sensor sets from [7] (Figure 2B).

We notice many similarities between the two figures: (1) The distribution of the colours is very similar; (2) Sensor 9, which was the most ‘popular’ sensor in the feature sets from [7] has almost the identical percentage of appearance in Figure 2A; (3) Sensor 7 was rarely used in both sets of selected features; (4) In Figure 2B, Sensors 8 and 10 were preferentially used in the very small feature sets of only one or two sensors, while in our feature sets Sensor 10 was preferred in these size constraints with Sensor 8 preferred to a comparable but lesser degree.

Furthermore, in [7], the authors noted that “the best feature sets typically contain sensors of both the standard SnO_2_ and zeolite-coated CTO types” and 99.55% of the “sensor combinations that allowed 100% performance” use “sensors from both technologies”. This is validated by our observation in Table 1 where 90% of the selected sets contains sensors from both technologies. Note that this includes sensor size constraint 1 for which the criterion obviously cannot be fulfilled. If we exclude these cases, we find that 97.89% of the remaining selected sets contain sensors of both types. When only one type of sensor was used in classification in [7], the SnO_2_ sensors have much better performance than the CTO sensors. This also agrees with the results in Table 1 where (a) SnO_2_ sensors were utilised more often than the CTO sensors in most optimal feature sets, and (b) on those occasions where only one sensor type was used, SnO_2_ sensors were chosen 90% of the times.

The similarities described indicate that the feature sets discovered here match closely those found by exhaustive search of classification results.

While the overall patterns between the two figures are similar, there are also some subtle differences in the frequency of appearance of the sensors as identified by the two methods: (1) In [7] Sensor 9 was included in almost all feature sets for size constraints of 5 or more sensors, whereas in our case it is used less often (90% of selections) and Sensor 1 assumes the dominant position. (2) For feature sets of small size constraints, Sensor 8 was utilised over 50% of the times in [7], while here Sensor 10 was picked more than 80% of the times. Further, Sensor 1 and 6's appearances for size constraint of 1 are swapped. (3) The percentage of appearance of sensors 10 and 11 in the optimal feature sets seems to almost switch around between the two methods, apart from those with very small size constraints. (4) Sensor 4 was utilised a lot less in the results here than in the “top ten” sensor sets in [7]. The reasons for these differences are not obvious but it is interesting to note that sensor 1 has the highest sensitivity within the group of CTO sensors, even though its signal-to-noise ratio (SNR) is not particularly good within the group of CTO sensors and much worse than any of the SnO_2_ sensors. Sensor 9, on the other hand, has sensitivity at the lower end of the SnO_2_ group, albeit much higher than any of the CTO sensors. Furthermore, sensor 4 has by far the lowest sensitivity of any sensor even though it has the highest signal-to-noise ratio within the group of CTO sensors. This dichotomy may be behind the differential preference for the readings of this sensor in the MI and wrapper feature selection. In other words, the MI method has a preference for sensors with high sensitivity (resulting in high variances in the data), while the wrapper approach prefers those with high SNR. This difference in preferences of selected sensors could be due to the linearity of the SVM classifier and the non-linear nature of the Kraskov-Grassberger technique we employed in estimating the probability distribution of the data (see Methods).

Having compared the prevalence of sensors and features in the chosen feature sets we now proceed by examining the classification results reported in [7] for the features sets that we here selected via the maximum MI criterion. Figure 1A shows the classification performance (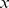-axis) of all the different feature sets against the size constraints (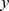-axis), while the frequency with which each feature set appears in a size constraint is shown by the colour as indicated by the colour bar. Figure 1B shows the same results for those with classification performance of greater than 0.96. For comparison, the classification performance of all the permutations of feature sets is shown in Figure 5.

When comparing the two sets of results, we observe that the feature sets selected by the maximal MI criterion give close to the best achievable classification results. All the classification results from these selected feature sets are above the 75% quantiles shown in Figure 5. For example, for the size constraint of 1 sensor and 1 data point, the 75% quantile is 0.6 with the best result being 0.7, the performances in Figure 1 for the constraint are between 0.62 and 0.7. Further, we can see in Figure 1 that the classification result of the majority of the optimal feature sets selected were very close to the top performances. In other words, the maximal MI criterion was able to identify those features that give close to optimal performances in the classification. This is important, since as a filter technique it is much more efficient at feature selection than the exhaustive search of a wrapper method.

Now, we note that while these feature selections did not necessarily give the very best possible classification performance (e.g. many of the results in the “best” column in Figure 5 have a performance of 1), the difference in performance is actually only about 1 or 2 errors, which is not statistically significant for a data set of 200 samples. Second, it is important to remember that the best performances reported in [7] are not a realistic prediction of what a wrapper approach to feature selection would achieve for truly new data. A first indication of this is that rerunning the classification using the “top 10” feature sets with different training/test set splits in [7] already resulted in slightly worse performance in general. A true estimate of the performance of a classifier system with a wrapper approach would only be obtained if the wrapper method was completed with training and evaluation sets and the features so identified would then be tested with separate testing data. Our comparison to the work in [7] should hence be seen as a comparison to the theoretically best achievable performance (as identified by the extensive search of a wrapper method) rather than a comparison to a wrapper method. As such, we conclude that the maximal MI criterion performs as well as one could expect from a filter method.

**Figure 6:**
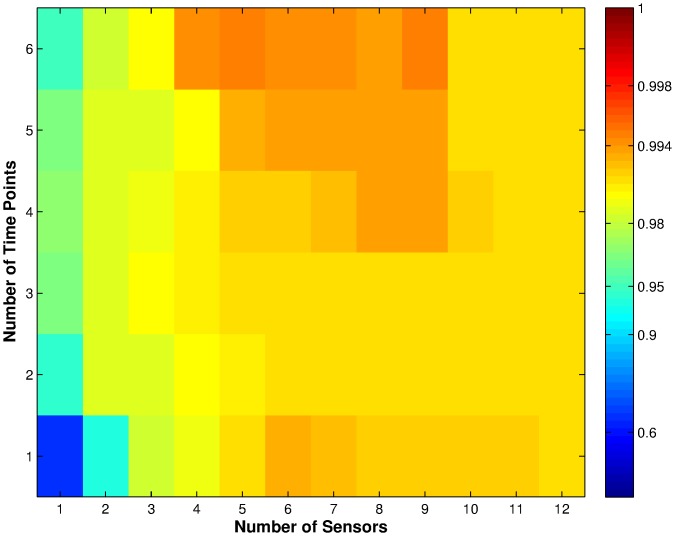
Average classification performance from [7] using the feature subsets identified by MI, weighted by the number of times each set was chosen in the training sets. Each cell in the figure represents a number of sensors (x-axis) vs. time points (y-axis) combination. The colours in the cell represent the classification performance as shown by the logarithmic colour scale. High performance levels, close to unity, are resolved into many colour gradations from dark red to cyan. Weaker performances, below 0.9, are compressed into a few shades of blue.

Figure 6 shows the average classification results from [7] of all the optimal feature sets identified via the maximal MI criterion, weighted by the number of times each set was chosen in all the training sets. Comparing these results with the “top 10” column in Figure 5, we can again see that the average performance is not necessarily the best possible (as shown in the “top 10” of [7]), however as described above it is very close, only differs by 1 or 2 errors. Further, each average classification performance in Figure 6 resulted from between 1 to under 20 different feature sets, thus for most size constraints Figure 6 covers more classification performances than Figure 5. Lastly, as the training sets are fairly small, selection bias in some partitions of the ten-fold validation process will see features picked that are only optimal to that training set.

### Classification Results

Thus far we have focussed on linear support vector machine classifiers as in [7]. In this section, we compare the results obtained using some other common classifiers. Each of the 100 test sets (10 repetitions of ten-fold cross validation) are examined by the classifiers using the optimal feature set determined by the maximal MI criterion for the corresponding training set, the observed performances are averaged and reported below.

**Figure 7:**
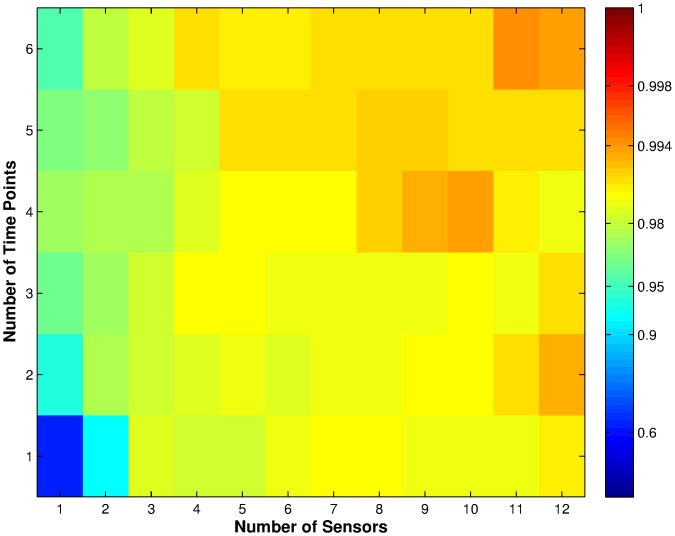
Average classification performance for all the test sets using linear SVM with C = 65536, weighted by the number of times each set was chosen in the training sets. Each cell in the figure represents a number of sensors (x-axis) vs. time points (y-axis) combination. The colours in the cell represent the classification performance as shown by the logarithmic colour scale. High performance levels, close to unity, are resolved into many colour gradations from dark red to cyan. Weaker performances, below 0.9, are compressed into a few shades of blue.

Before comparing the classifiers' results, we first compared the results in Figure 6 with SVM classification results of the test sets used in this paper (Figure 7). The results in Figure 7 were obtained using linear SVM with cost parameter 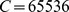, which is the same as the method used in [7]. Comparing the two sets of results we can see that there are slight differences in performance. This is not surprising, as the classifications were performed on differently partitioned training and test sets, which can give different results [7]. The difference, however, is so slight that it is statistically insignificant for the small data set used here.

**Figure 8:**
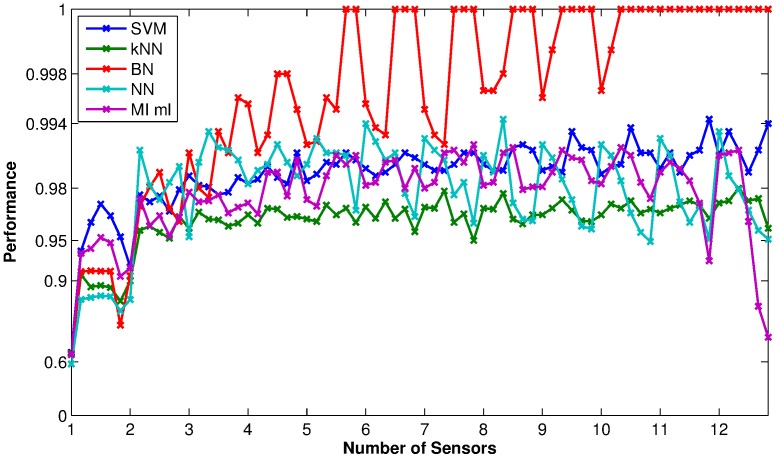
Comparison of performance among some common classifiers using feature sets selected by the MI criterion. The x-axis shows the sensor size constraints used in the feature selection, each tick mark represents the time point size constraint of 1, the performance values as marked out on the plots between two x-axis tick marks represents time point size constraints 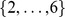. The y-axis shows the performance in a highly non-linear, logarithmic scale the same as the colour bars for Figures 6 and 7 for easy comparison.

Figure 8 shows a comparison of classification results among a number of the different classifiers – support vector machine (SVM), k-nearest neighbours (kNN), Bayesian networks (BN), neural networks (NN), mutual information maximum likelihood (MI ml). All classifiers performed consistently well for sensor size constraints of more than 2, and there was little actual difference in the overall accuracy (note the strongly non-linear scale on the y-axis). We see from the figure that the Bayes Network classifier has consistently high accuracy when the size constraint allows a large number of sensors. To test whether the differences in the classification performances are statistically significant, we performed two-tailed 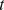-test (see Methods, Equation 2) to compare the performances at each size constraint between any pair of classification methods. We found that, for most of the size constraints, the BN classifier's performance was superior to that of the kNN classifier with a p-value 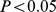. For some size constraints the BN classifier's performance is also significantly superior to the performance of either NN or MI classifiers. There was effectively no statistically significant difference in performance between BN and SVM classifiers.

**Figure 9:**
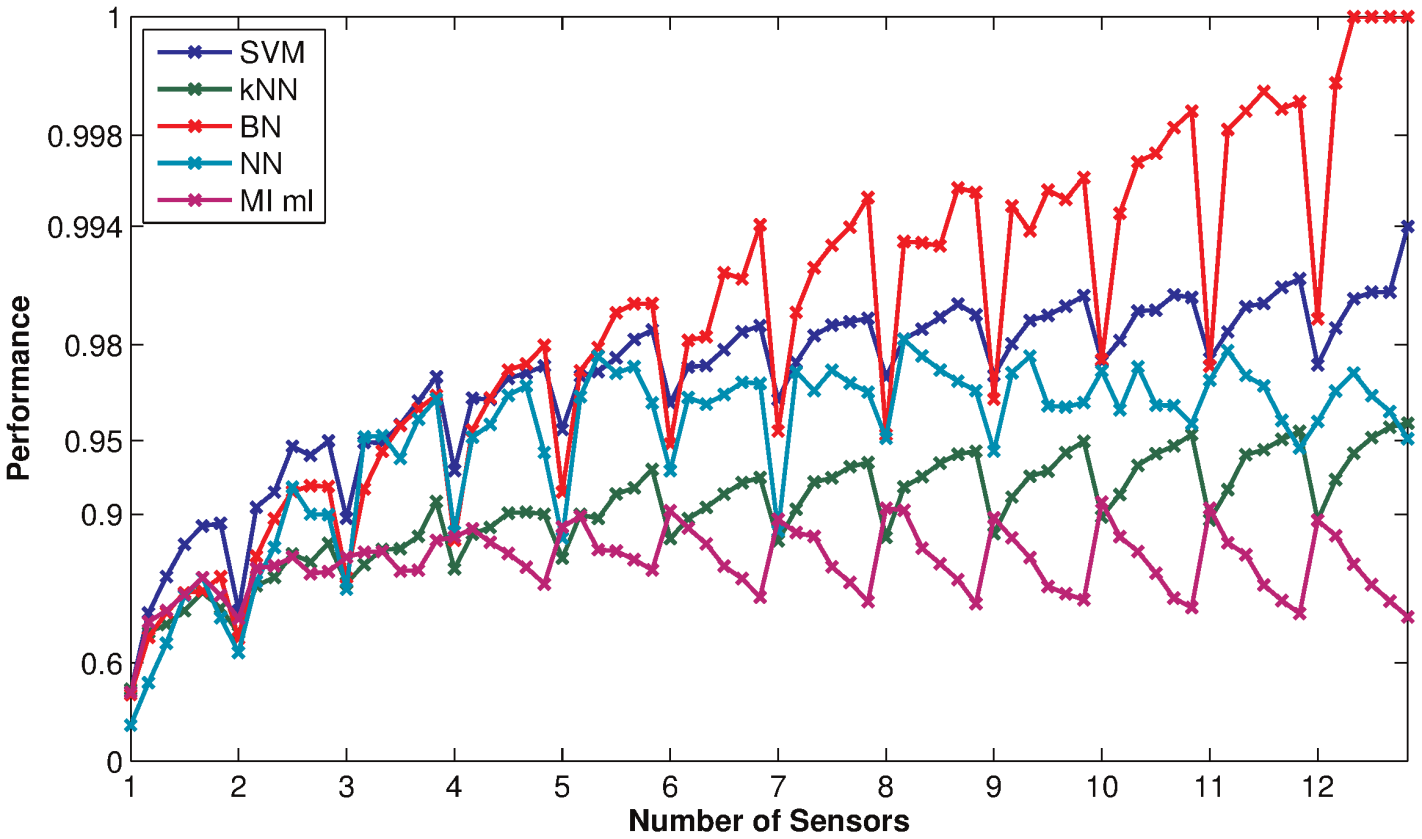
Classification results of the data set from some common classifiers using randomly selected features. The x-axis shows the sensor size constraints used in the feature selection, each tick mark represents the time point size constraint of 1, the performance values as marked out on the plots between two x-axis tick marks represents time point size constraints 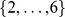. The y-axis shows the performance in a highly non-linear, logarithmic scale the same as the colour bars for Figures 6 and 7 for easy comparison.

For all the classifiers examined and for each size constraint, we then compared the performance of the feature sets selected using the MI criterion against 100 randomly selected feature sets. Figure 9 shows the average classification results on the data sets for all the methods using the randomly selected features. Comparing the two figures, we can see that the classification performances using features selected by MI are consistently higher than those using randomly selected features, at all size constraints. Using a two-tailed 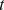-test (see Methods, equation 2), we found almost all differences in the performance between MI-selected and randomly-selected feature sets are statistically significant at 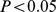. Therefore, the consistency in the better classification performance using the features selected by the MI method across all classification methods shows that the feature selection algorithm was able to select the subset that was advantageous to the employed classifiers.

## Discussion

### Comparison to MI with no constraints

We formulated the feature selection task by imposing separate constraints on the number of sensors and the number of time points in the feature set. A chosen feature combination then uses the same selected time points for each of the selected sensors. This constraint on the candidate feature sets greatly reduces the number of features in the search space, for example, there are 
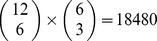
 combinations for 6 sensors and 3 time points with this constraint, compared to 
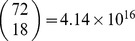
 combinations for an arbitrary combination of 18 features when this constraint is not imposed. However, it is worth noting that the imposed constraint implies that the feature set with the truly maximal MI might not be found.

**Figure 10:**
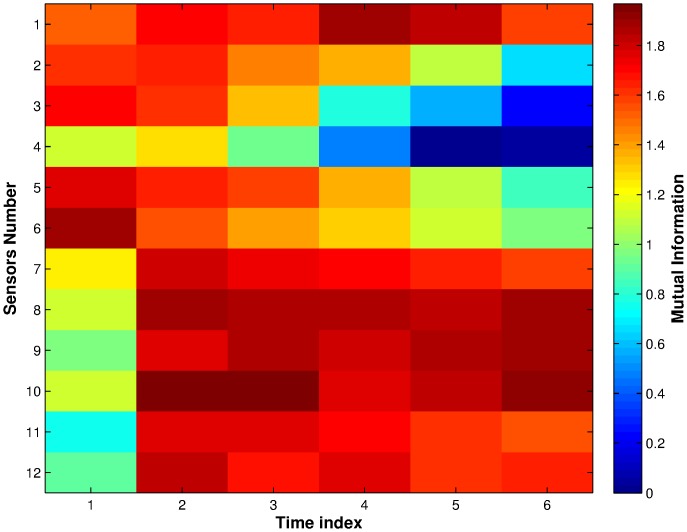
Mutual information (MI) between individual features and the class. Note that sensors 1–6 were CTO sensors and 7–12 were SnO_2_ sensors.

Figure 10 shows the MI between the individual features and class for the entire data set. From this figure, we can see that the SnO_2_ sensors have higher MI with the class than the CTO sensors in general. This is consistent with the frequency of appearance of sensors and features in Figures 2A and 4, where the SnO_2_ sensors were selected more often than the CTO sensors. Further, it can be seen that sensor 10 has higher MI values than all other sensors; this is consistent with Figure 4, where sensor 10 is chosen more often than not when there is a constraint of just one sensor. An example of a feature not chosen due to the constraints is Sensor 6 at time index 1, which has high MI in Figure 10, however, both Figures 2A and 4 show Sensor 6 is not picked often. This is because neither the rest of time indices in Sensor 6, nor the other sensors in time index 1 have high MI. While Sensor 6 at time index 1 does have high MI, it does not appear to provide enough new information in addition to multiple time indices from other sensors to result in it being selected.

Removing the feature selection constraints may choose features that would not be included when constraints are imposed, as discussed above. However, when considering the question of sensor and feature selection for the purpose of manufacturing a device, the use of size constraints, at least on the sensors, is advantageous when the cost of manufacturing needs to be considered. Further, from the comparison of individual MIs shown in Figure 10 with the frequency of appearance of features in Figure 4, we expect that for our dataset many of the same features will be selected when no constraints are placed. A full comparison of feature selection and classification results without constraints is beyond the scope of this paper and will be explored in future research. Crucially, note that due to the combinatorial explosion when no constraints are in place, the wrapper method will be intractable, and even the filter method will require smarter exploration or approximations (e.g. mRMR or QPFS) of the full feature space.

### Comparison with PCA Analysis

**Figure 11:**
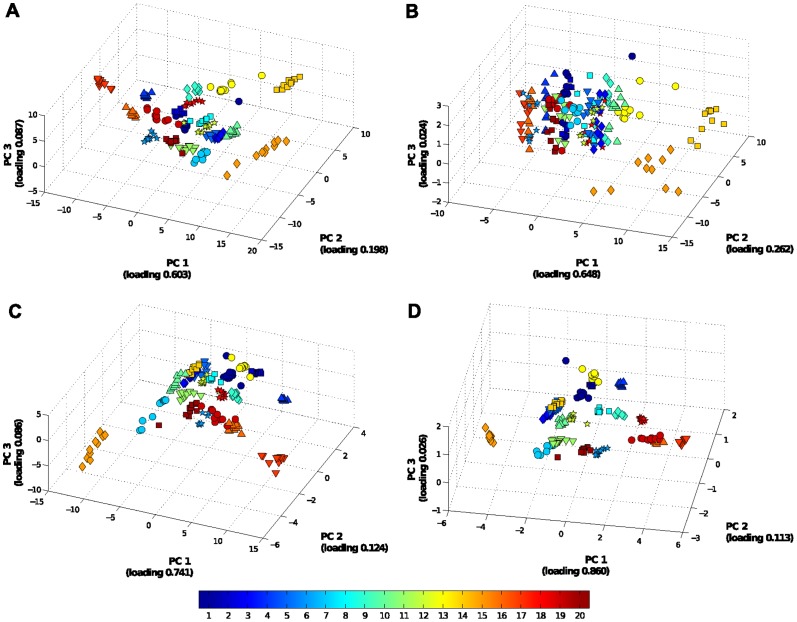
PCA plots of the data set. (a) all sensors; (b) all CTO sensors; (c) all SnO_2_ Sensors; and (d) Sensor 10 only. For all plots, all the time points are used. The class numbers are coloured according to the scale shown.

Figure 11 shows the first three principle components of the data set for all sensors, for the CTO sensors only, for SnO_2_ sensors only, and for data from sensor 10 only, which is the sensor with very high MI with the classes (Figure 10). The principle components were calculated based on the covariance matrix of the data. The PCA plots agree in principle with the frequency of appearance of sensors in Figure 2, that is, the SnO_2_ sensors have better discrimination capability on this data set than the CTO sensors. There are several reasons for this discrepancy in classification: Firstly, as Figure 3 shows, the absolute response of CTO sensors (maximum around 0.2) are a lot lower than the SnO_2_ sensors (maximum around 0.9). The responses of CTO sensors with coating (sensors 2–5, Table S1) are in general lower than 0.1, which is close to the natural response of the sensors to dry zero grade air used as a carrier gas for the chemical samples and recovering phase (see Methods). Secondly, the CTO sensors were not specially made to respond to these particular chemicals, while the SnO_2_ sensors do have the capability for detecting a large range of organic chemicals (see Table S2).

Further, comparing the PCA plots of all sensors and the SnO_2_ sensors, we see that the first three principle components of the data using all the sensors forms better clusterings than those using SnO_2_ sensors only. This also confirms the finding in Figure 2 and Table 1, where a combination of sensors from both types is often selected in the feature sets. Lastly, the PCA plot of the data using sensor 10 only, agrees with the high MI result of Figure 10 and the frequent appearance of the sensor in the selected feature sets in Figure 2A because even though this is using only one sensor, the classes are still reasonably separated.

## Conclusion

We approached the problem of feature selection for arrays of metal oxide sensors in classification with constraints on the combination of number of sensors and time points for each feature set by using a filter approach to maximise the mutual information among the selected features and the class (identity of chemicals). We found that our method allows us to discover features that give consistently better classification performances than randomly selected features across all the classifiers tested. Furthermore, the classification performance was not significantly different from that previously observed in an exhaustive search of all possible feature combinations (within size constraints) with an SVM classifier on the same data. The results described here were achieved with a computational burden approximately three orders of magnitude lower than what was needed for the exhaustive search that would form part of a wrapper approach. We also note in passing that MI may be seen as a computational implementation of the concept of “sensor independence” [37], which has been suggested as a useful comparator of the performances of different chemical sensor arrays.

Notwithstanding these encouraging findings, it has already been show that this data set permits highly accurate classification results for many of the possible feature sets. In future work, we will test the MI feature selection approach with more challenging data sets obtained, for example with multiple concentrations of chemicals and taken over an extended period of time (e.g. [12], [38]) to incorporate the detrimental effects of sensor drift in the data, or indeed more noisy and less easily classified samples taken in real world applications.

## Materials and Methods

### Electronic Nose Measurement

The electronic nose, its measurements and the data used in this paper are exactly the same as those in [7]. We will describe these details in brief here for completeness.

The equipment used here was the FOX 3000 Enose (Alpha M.O.S., Toulouse, France) with two arrays of semiconducting sensors – six chromium titanium oxide (CTO) specially manufactured for this testing and six standard doped tin oxide sensors (SnO_2_) originally equipped in the instrument. For the six CTO sensors used here, five were coated with zeolite [39] and one uncoated. The coating of CTO sensors adds a transformation layer, which is designed to modify or restrict the composition of gases that comes into contact with the sensing element underneath. In this case, zeolite is used due to its porous nature, having pore and channel structures of molecular dimensions. By controlling the pore size and permeability, the zeolite coatings are able to restrict the size and shape of gas molecules reaching the sensing element. Table S1 [7] gives a list of the specific sensors, their sensor identity in the measurement, and a short description of the composition.

Twenty different chemical compounds (classes) were analysed using the FOX eNose. The compounds are from four chemical groups: alcohols, aldehydes, esters and ketones, each with five chemicals (see Table S2 for details). The chemicals were chosen from a large set of chemicals used in a comparison of metal oxide with biological sensors [37]. Each sample was diluted using paraffin oil to give final concentrations in the range of 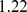 and 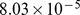 M (Table S2). For each chemical, 10 replicates of the samples were prepared, giving us a total of 200 samples analysed.

The chemical samples were analysed over 4 days. Samples of 1 ml were presented in a 20 ml glass vial using the static headspace method. The instrument was equipped with an autosampler (HS50, CTC Analytics, Switzerland), which allows reproducible injections. The headspace volume of 500 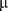
l of each sample was taken for analysis, and samples were analysed in groups based on chemical family. Dry zero grade air was used to sweep the sample through two chambers housing the different types of sensors. For each sample, the sensor records 300 s worth of data at 2 Hz. The instrument is then flushed with zero grade air for 240 s to allow the sensors to return to baseline. Figure 3 shows the response of the sensors towards a chemical compound. The data were captured and pre-analysed using AlphaSoft v.8 (Toulouse, France). (See Dataset S1 for full data set.)

### Feature Set

The full feature set used in this paper is also the same as those in [7]: For each sensor, 6 candidate time points were extracted, those were: 10, 20, 30, 40, 50 and 60 seconds (cf. Figure 3) from the full data taken over 300 seconds at 2 Hz. Therefore, the full feature sets comprised of these 6 time points from each of the 12 sensors. This gives 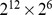 total permutations of combined sensors and time points. For example, for 6 sensors and 3 time points, there are 
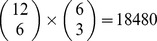
 combinations.

### Cross-validation

All results reported were obtained from 10-fold balanced cross-validation as described in [7]. The full data set was partitioned into training and test sets by randomly selecting 10% of the measurements from each chemical to form the test set, and the remaining to form the training set. This process was performed ten times, where each data sample appears exactly once in a test set. We then repeat this entire procedure ten times to gather 100 test (and their corresponding training) sets.

### Optimal Feature Selection

The optimal feature selection task is to select a subset of features, 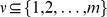, where 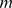 is the total number of features, such that the resulting subset of features gives the best classification performance for the given size constraint 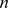 on the number of features in 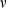. We select the subset of features by maximising the *mutual information* 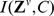 [30] between the selected features 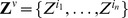 and class 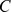:
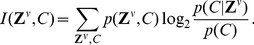
(1)This approach was suggested by Battiti [31], since it minimises the uncertainty (entropy) 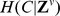 about the class given the features. MI is increased with the independence of information that the features contain about the class (similar to the concept of “sensor independence” in [37]). However, the MI between feature and class is increased by only the component of independence of the features which is relevant to the class. That is, MI is not increased where features contain redundant information about the class. Furthermore, MI captures the synergies between the features, that is, what multiple features when considered together (but not separately) can reveal about the class. Further discussion of the nature of redundancy and synergy in MI can be found in [40]. A subtle point is that maximising MI does not mean eliminating all redundancies between the features — it does mean any redundancies in the features *about the class* are not double-counted, and any features which carry redundancies are only selected due to unique and synergistic information they provide.

Ordinarily we would evaluate 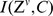 for all 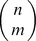 combinations of 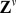 for a given number 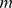 of features. Whilst this is inefficient, and there are alternatives to evaluating all combinations (e.g. the greedy forward feature selection approach described by Battiti [31]), evaluating all combinations here allows us to evaluate the features selected by mutual information against all possible feature selections. Also, note that in this paper our feature selection is not constrained simply to a subset of size 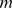 of the total number of features, but to select 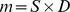 features, where 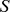 is a given number of sensors, 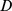 is the number of data points (measurements in time) per sensor and the same 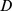 times must be selected for each sensor. This means we have 
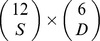
 combinations of features to evaluate.

Another challenge in evaluating Equation 1 here is to estimate the multivariate joint and conditional density functions for a small data set (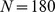 for each training set). Early approaches tried to get around the issue by using pairwise approaches (e.g. by Battiti [31], and the mRMR [34] and QPFS [35] methods). However, pairwise approaches are effectively heuristics; they do not directly remove redundancies between features *about the class* specifically, and they provide no mechanism to capture synergistic information provided by multiple features about the class. Other possibilities to act against small data set size include adaptive forward feature selection, e.g. [33], which we will investigate in later work. In this paper, we employed the Kraskov-Grassberger technique [36] (estimator 2) for estimating the the mutual information. This technique uses dynamic kernel widths and bias correction, which provide robustness against small data size. The code used for the estimation is the publicly available Java Information Dynamics Toolkit [41].

### Classification Methods

We used several common classifiers to evaluate the effectiveness of the optimal feature selection.

A **support vector machine** (SVM) [42] constructs a hyperplane to separate training data in different classes. We used the libsvm library [43] to perform the classification using C-SVC (SVM classification with cost parameter of 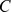). Nine 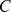 values, starting with 1 and increasing by a factor of 4 for each subsequent value, were tested (i.e. 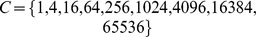) to capture the classifier's performance over large range of 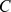 values. Two types of SVM were used: linear and a nonlinear SVM using Gaussian radial basis kernel function. The Gaussian radial basis kernel function is, 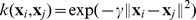, where 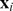 is the vector of the data for sample 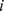 and 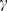 is the kernel width. We selected 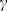 in two ways: the inverse of number of features (the default 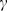 value in libsvm) and the inverse of the 0.1, 0.9 quantile and the median of pairwise distances of the data (See Figures S2, S3, S4 for the distribution of pairwise distances, and Figure S5 for a comparison between the two measures). We found in all cases, 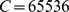 performs better than or consistently with the other 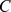 values (Figures S1, S6, S7, S8, S9, S10, S11, S12). Moreover, we found consistent performances between linear SVM and radial SVM with 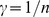, both better than the classification performances of radial SVM with other 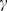 values (Figure S13). Therefore, we use the classification performance of linear SVM with 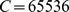 in comparison with the other classifiers. To ensure that this result is optimal, we further performed linear SVM with 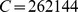 (i.e. another 4 fold increase on the final 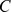 value) and found this does not improve the classification performance (Figure S1).

The ***k*** **nearest neighbour** (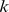
NN) algorithm compares the input data with an existing set of training data by computing a distance metric [44]. The neighbours of the input data are the 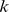 data points with the smallest distance metric. The input data's class is determined to be that with the most data points in the neighbourhood. In this paper, we used 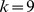, this is the number of data samples each class has in the training set, thus the most any class can appear in the neighbourhood of the input data.

In **Bayesian Networks** (BN) [45], each node represents a random variable (these could be observed data or latent variables), 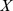, with associated probability functions, 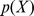. The nodes in a BN are linked by directed edges which indicate the conditional dependency between the variables, for example, if the nodes 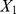 and 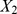 in a BN are linked by a directed edge from 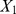 to 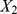, then 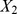 is dependent on 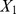 and 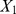 is the *parent* node to 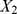. Lastly, the network as a whole is acyclic. We used the BNT toolbox [46] to implement the network and perform the classifications. Three network structures were implemented, a Naïve Bayes Network where a node representing the class of the chemicals is parent to all other nodes each of which represents a feature, and two other networks which are modifications on the Naïve Bayes Network. The modified networks are: (a) each node, 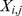, represents a feature and has parents (i.e. it is dependent on) the nodes 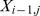 and 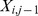, where 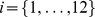 is the sensor number and 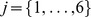 is the time point number as described in subsection on Feature Set; (b) each node 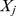 represents all sensors at time point 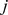 and has parent the node 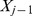. In all three networks, only the features selected by the MI measure were included in the network. We found the Naïve Bayes networks performs not as well as the modified networks, but there is no significant difference between the performances of the modified networks. We report here results from the modified network where each node represents a feature. The sensor numbers for this modified network were those shown in Table S1 and Figure 3.

An artificial **Neural Network** (NN) [47] consists of a group of nodes, or neurons, that are interconnected. We used here a feedforward NN called multilayer perceptron (MLP) to map the feature data (input neurons) onto the chemical classes (output neurons) through two layers of hidden neurons. The MLP was implemented using the Netlab toolbox [48] with maximum of 100 iterations using three optimisation algorithms (quasi-Newton, conjugate gradients, and scaled conjugate gradients) and 6 or 12 hidden nodes. We report here results from MLP with 12 hidden neurons using quasi-Newton optimisation, which performed slightly better than the others.

**Mutual Information maximum likelihood** makes a classification as the class 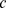 which maximises the local or pointwise mutual information 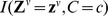 with the observed (selected) features, where the pointwise mutual information is 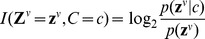 [49]. This approach is equivalent to selecting the class which maximises the probability 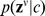 of the observation given the class, and is also equivalent to a classic maximum likelihood classifier (maximising the posterior 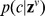) under the assumption of the marginal distribution for each class 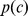 being equiprobable [50]. The mutual information here was evaluated using Kraskov-Grassberger estimation [36] (i.e. we do not assume a multivariate normal distribution here), using the Java Information Dynamics Toolkit [41].

### Hypothesis Test

The two-sided student's **t-test** is used to test the null hypothesis that there is no difference between the classification performance of two classifiers or two sets of data. When the hypothesis is true and the population is normal, the test statistic 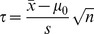 has the Student's 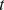 distribution with 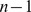 degrees of freedom (i.e. 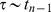), where 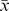 and 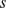 are the mean and standard deviation of the classification performance of features selected by MI criterion, 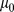 is the mean of performance using randomly selected features, and 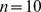 is the number of repeats of ten-fold cross-validation performed. The p-value (for two-sided test) is [51]:
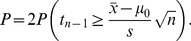
(2)


## Supporting Information

Figure S1
**Average classification performance for all the test sets using linear SVM with different cost values.** The x-axis shows the sensor size constraints used in the feature selection, each tick mark represents the time point size constraint of 1, the performance values as marked out on the plots between two x-axis tick marks represents time point size constraints 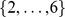. The y-axis shows the performance in a highly non-linear, logarithmic scale.(TIF)

Figure S2 **Histogram of 0.1 quantiles of the distribution of pairwise distance for all possible feature choices.**
(TIF)

Figure S3 **Histogram of median of the distribution of pairwise distance for all possible feature choices.**
(TIF)

Figure S4 **Histogram of 0.9 quantiles of the distribution of pairwise distance for all possible feature choices.**
(TIF)

Figure S5 **Comparison of inverse of number of features and the median pairwise distances for all possible feature choices.**
(TIF)

Figure S6
**Average classification performance for all the test sets using radial SVM with different cost values and** 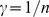 **, where** 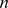 **is the number of features, for the kernel function.** The x-axis shows the sensor size constraints used in the feature selection, each tick mark represents the time point size constraint of 1, the performance values as marked out on the plots between two x-axis tick marks represents time point size constraints 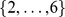. The y-axis shows the performance in a highly non-linear, logarithmic scale.(TIF)

Figure S7
**Average classification performance for all the test sets using radial SVM with different cost values and** 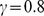 **for the kernel function.** The 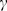 value used here is the inverse of the average of 0.9 quantile of all the pairwise distance of the data. The x-axis shows the sensor size constraints used in the feature selection, each tick mark represents the time point size constraint of 1, the performance values as marked out on the plots between two x-axis tick marks represents time point size constraints 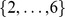. The y-axis shows the performance in a highly non-linear, logarithmic scale.(TIF)

Figure S8
**Average classification performance for all the test sets using radial SVM with different cost values and** 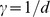 **, where** 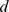 **is the 0.9 quantile of the pairwise distance of the given feature set size, for the kernel function.** The x-axis shows the sensor size constraints used in the feature selection, each tick mark represents the time point size constraint of 1, the performance values as marked out on the plots between two x-axis tick marks represents time point size constraints 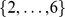. The y-axis shows the performance in a highly non-linear, logarithmic scale.(TIF)

Figure S9
**Average classification performance for all the test sets using radial SVM with different cost values and** 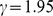 **for the kernel function.** The 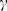 value used here is the inverse of the average of 0.5 quantile of all the pairwise distance of the data. The x-axis shows the sensor size constraints used in the feature selection, each tick mark represents the time point size constraint of 1, the performance values as marked out on the plots between two x-axis tick marks represents time point size constraints 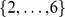. The y-axis shows the performance in a highly non-linear, logarithmic scale.(TIF)

Figure S10
**Average classification performance for all the test sets using radial SVM with different cost values and** 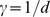 **, where** 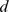 **is the 0.5 quantile of the pairwise distance of the given feature set size, for the kernel function.** The x-axis shows the sensor size constraints used in the feature selection, each tick mark represents the time point size constraint of 1, the performance values as marked out on the plots between two x-axis tick marks represents time point size constraints 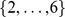. The y-axis shows the performance in a highly non-linear, logarithmic scale.(TIF)

Figure S11
**Average classification performance for all the test sets using radial SVM with different cost values and** 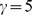 **for the kernel function.** The 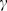 value used here is the inverse of the average of 0.1 quantile of all the pairwise distance of the data. The x-axis shows the sensor size constraints used in the feature selection, each tick mark represents the time point size constraint of 1, the performance values as marked out on the plots between two x-axis tick marks represents time point size constraints 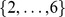. The y-axis shows the performance in a highly non-linear, logarithmic scale.(TIF)

Figure S12
**Average classification performance for all the test sets using radial SVM with different cost values and** 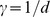 **, where** 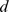 **is the 0.1 quantile of the pairwise distance of the given feature set size, for the kernel function.** The x-axis shows the sensor size constraints used in the feature selection, each tick mark represents the time point size constraint of 1, the performance values as marked out on the plots between two x-axis tick marks represents time point size constraints 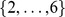. The y-axis shows the performance in a highly non-linear, logarithmic scale.(TIF)

Figure S13
**Comparison of average classification performance for all the test sets using linear SVM and radial SVM with different** 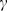 **values for the kernel function.** The cost value is set at 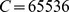, which is the 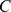 value with the best performance for each individual kernel function. The x-axis shows the sensor size constraints used in the feature selection, each tick mark represents the time point size constraint of 1, the performance values as marked out on the plots between two x-axis tick marks represents time point size constraints 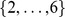. The y-axis shows the performance in a highly non-linear, logarithmic scale.(TIF)

Table S1 **Overview of the sensors used in the electronic nose.** A short description of the sensors is given here. CTO - Chromium-titanium oxide, SnO_2_ - Tin Oxide.
(PDF)

Table S2 **Chemical compounds analysed.** The chemical are shown here under their group names and corresponding concentration.
(PDF)
