# Supplementary figures and images for: Feature Selection for Chemical Sensor Arrays Using Mutual Information
Source: PLoS One. 2014 Mar 4;9(3):e89840. doi: 10.1371/journal.pone.0089840 (PMC3942325; doi:10.1371/journal.pone.0089840)

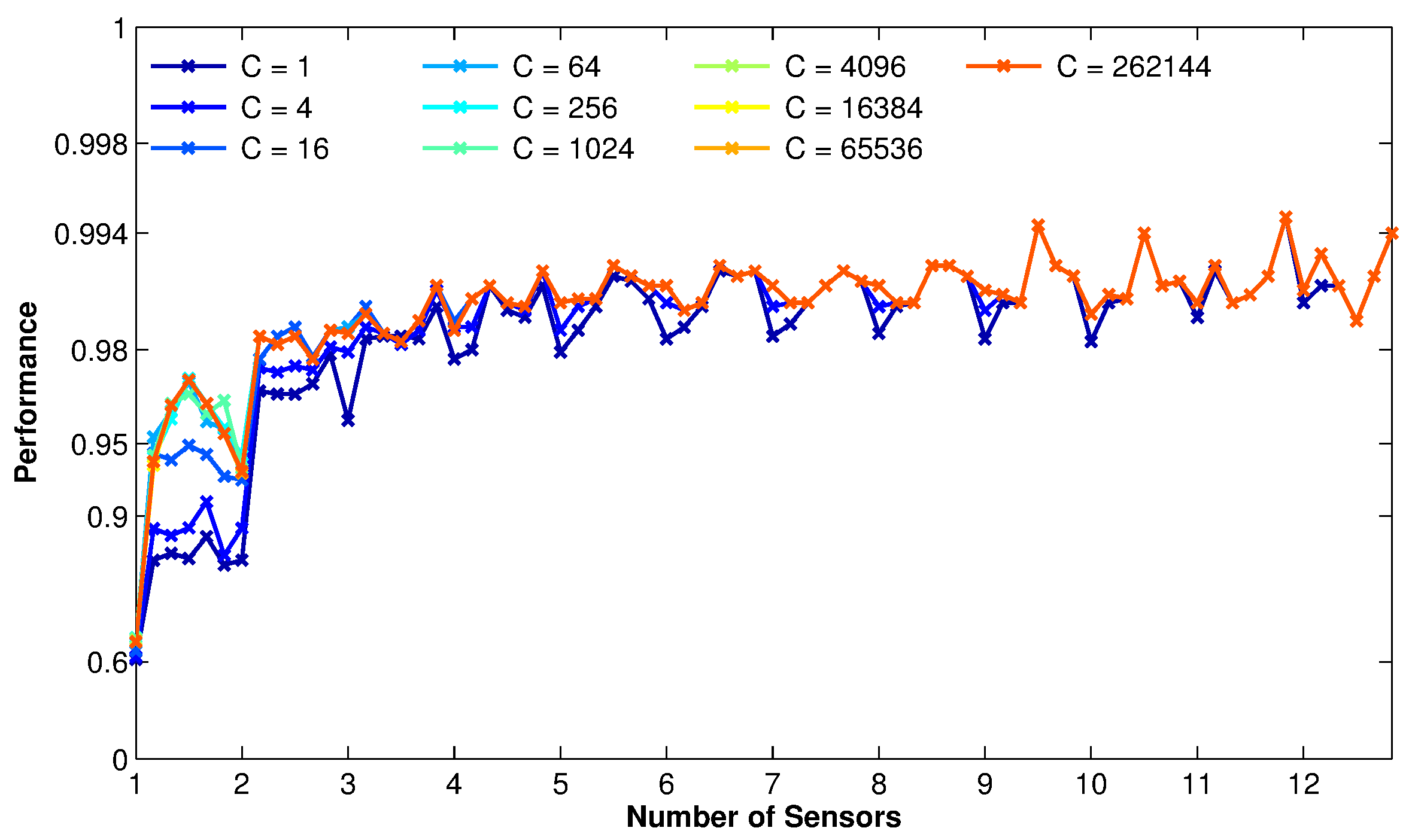

Supplement: Figure S1 — Average classification performance for all the test sets using linear SVM with different cost values. The x-axis shows the sensor size constraints used in the feature selection, each tick mark represents the time point size constraint of 1, the performance values as marked out on the plots between two x-axis tick marks represents time point size constraints . The y-axis shows the performance in a highly non-linear, logarithmic scale. (TIF) [file pone.0089840.s001.tif]

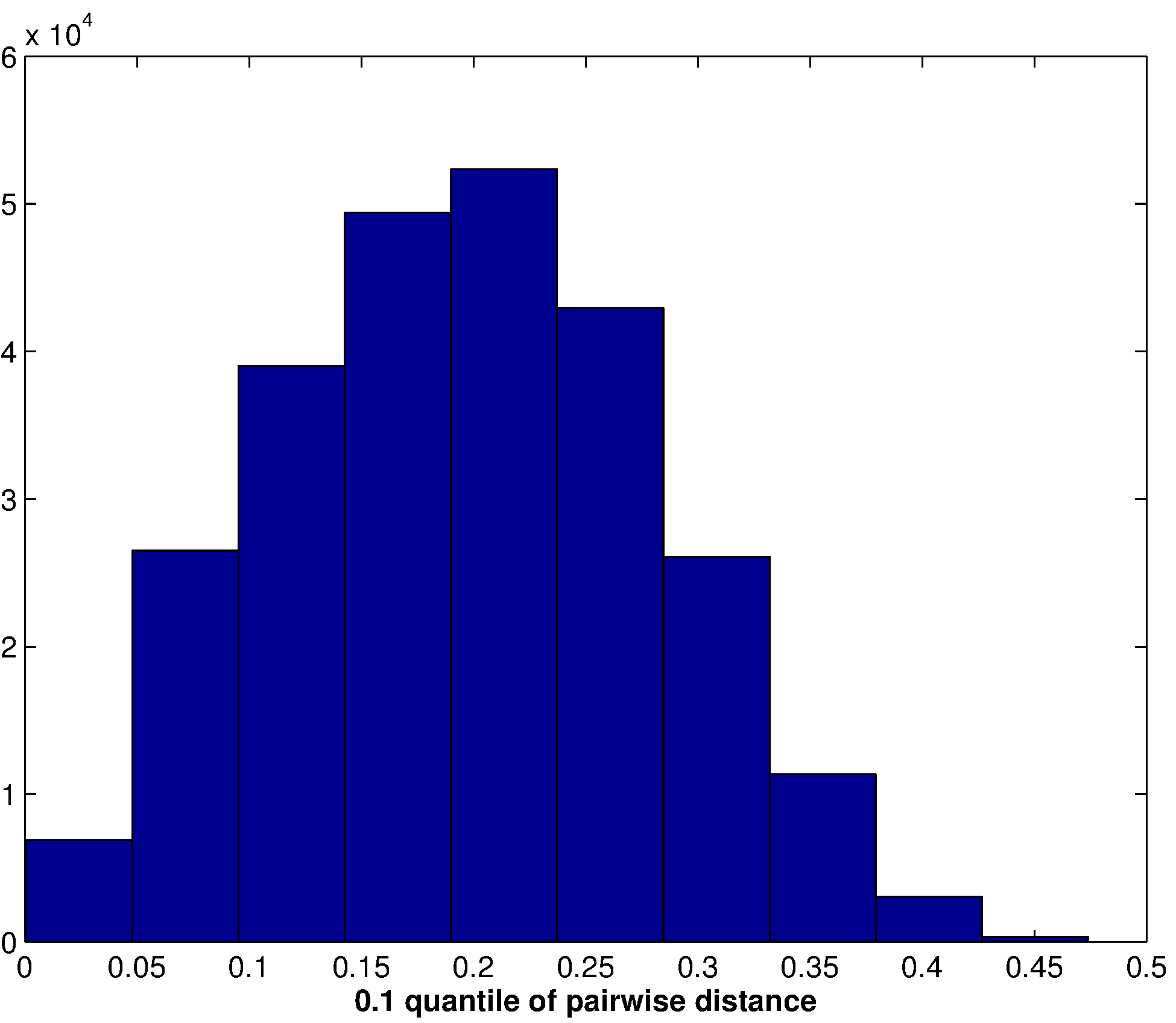

Supplement: Figure S2 — Histogram of 0.1 quantiles of the distribution of pairwise distance for all possible feature choices. (TIF) [file pone.0089840.s002.tif]

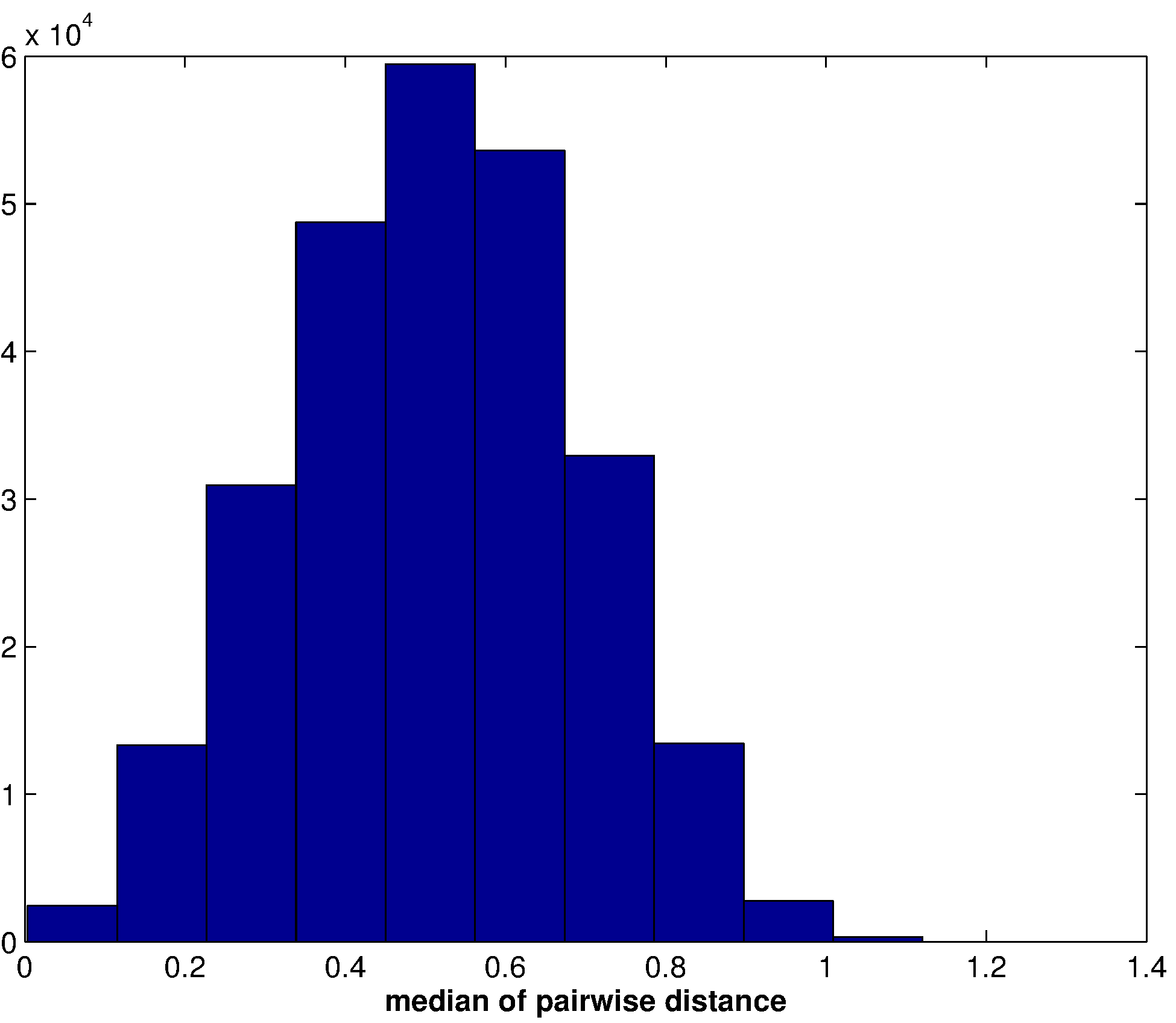

Supplement: Figure S3 — Histogram of median of the distribution of pairwise distance for all possible feature choices. (TIF) [file pone.0089840.s003.tif]

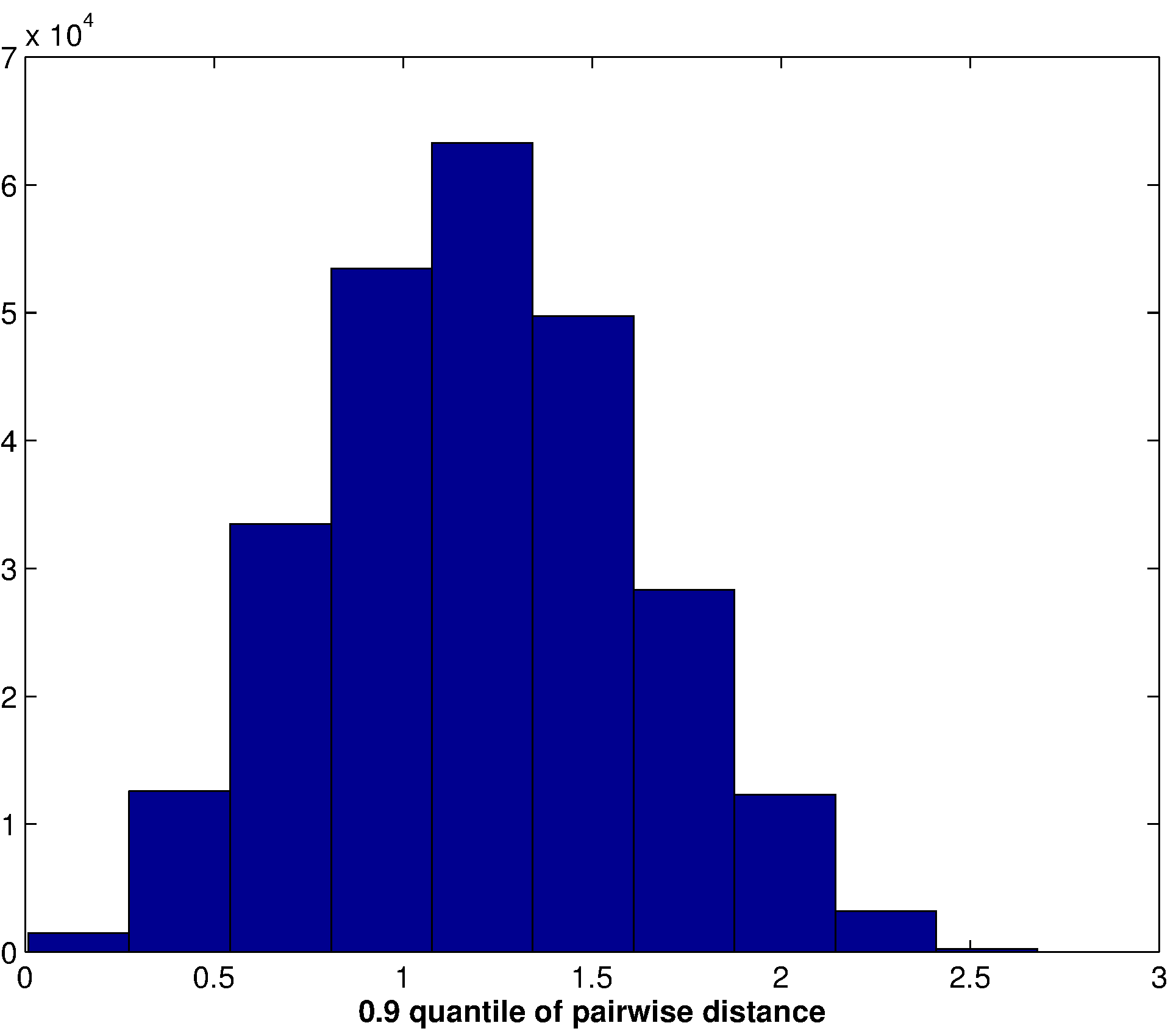

Supplement: Figure S4 — Histogram of 0.9 quantiles of the distribution of pairwise distance for all possible feature choices. (TIF) [file pone.0089840.s004.tif]

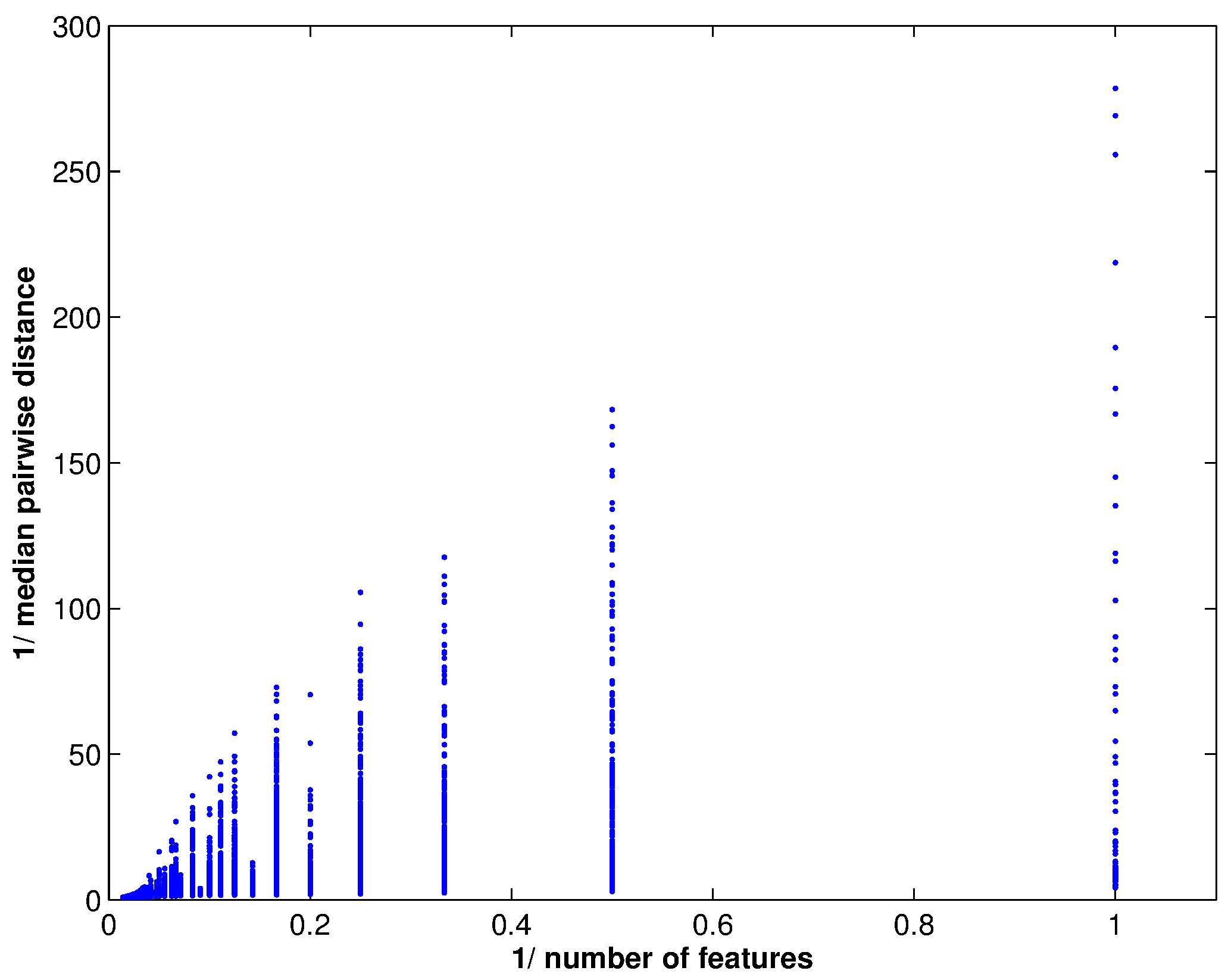

Supplement: Figure S5 — Comparison of inverse of number of features and the median pairwise distances for all possible feature choices. (TIF) [file pone.0089840.s005.tif]

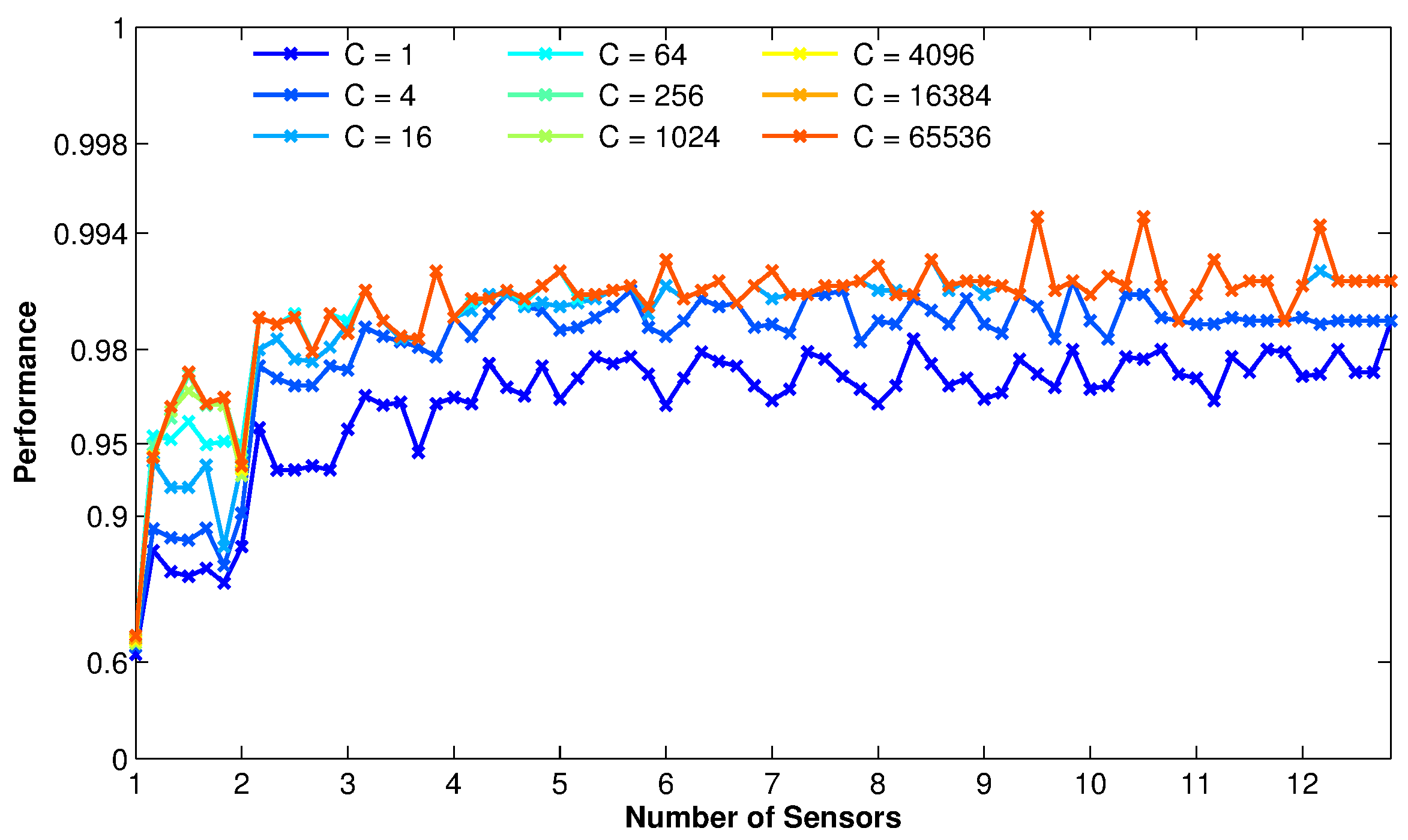

Supplement: Figure S6 — Average classification performance for all the test sets using radial SVM with different cost values and , where is the number of features, for the kernel function. The x-axis shows the sensor size constraints used in the feature selection, each tick mark represents the time point size constraint of 1, the performance values as marked out on the plots between two x-axis tick marks represents time point size constraints . The y-axis shows the performance in a highly non-linear, logarithmic scale. (TIF) [file pone.0089840.s006.tif]

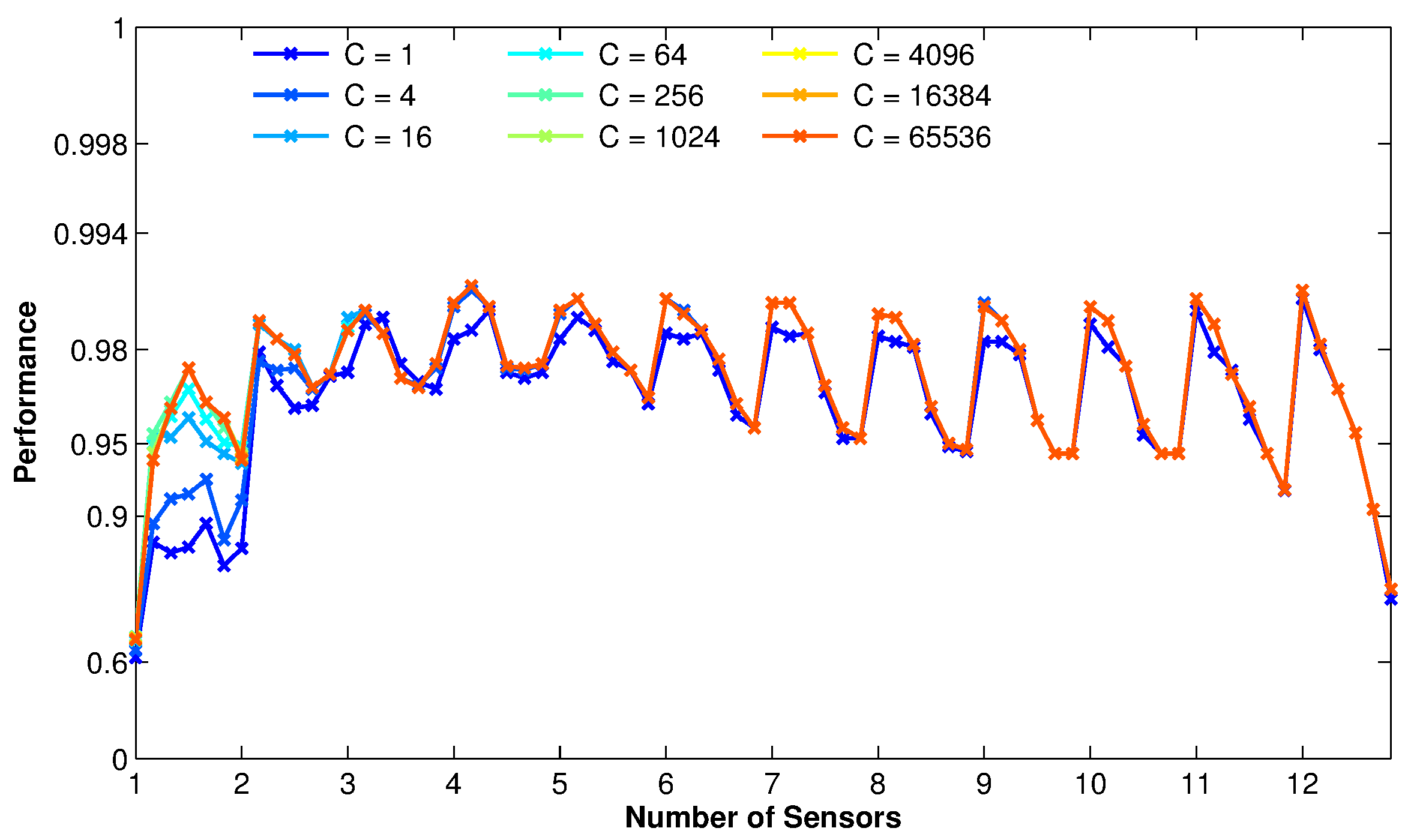

Supplement: Figure S7 — Average classification performance for all the test sets using radial SVM with different cost values and for the kernel function. The value used here is the inverse of the average of 0.9 quantile of all the pairwise distance of the data. The x-axis shows the sensor size constraints used in the feature selection, each tick mark represents the time point size constraint of 1, the performance values as marked out on the plots between two x-axis tick marks represents time point size constraints . The y-axis shows the performance in a highly non-linear, logarithmic scale. (TIF) [file pone.0089840.s007.tif]

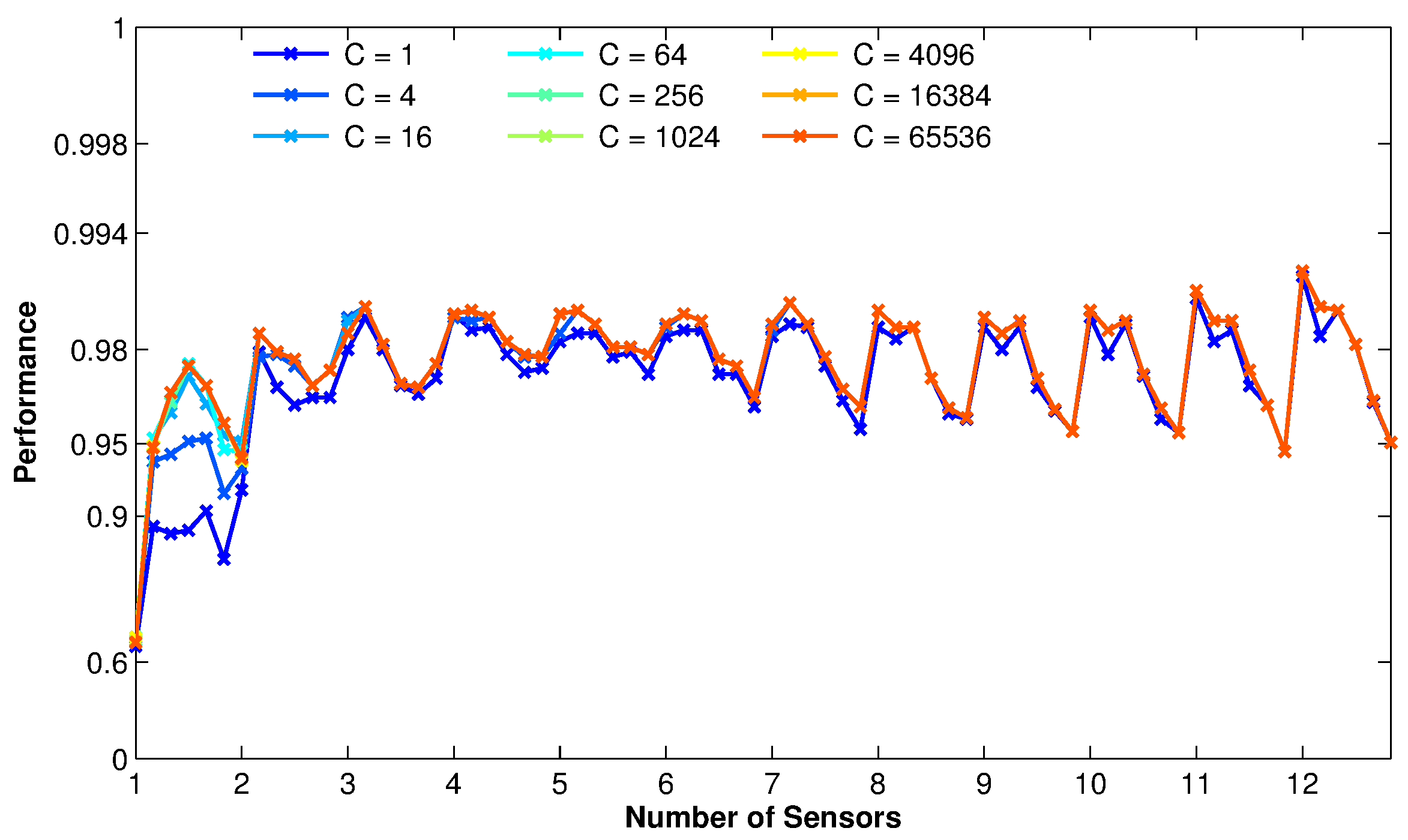

Supplement: Figure S8 — Average classification performance for all the test sets using radial SVM with different cost values and , where is the 0.9 quantile of the pairwise distance of the given feature set size, for the kernel function. The x-axis shows the sensor size constraints used in the feature selection, each tick mark represents the time point size constraint of 1, the performance values as marked out on the plots between two x-axis tick marks represents time point size constraints . The y-axis shows the performance in a highly non-linear, logarithmic scale. (TIF) [file pone.0089840.s008.tif]

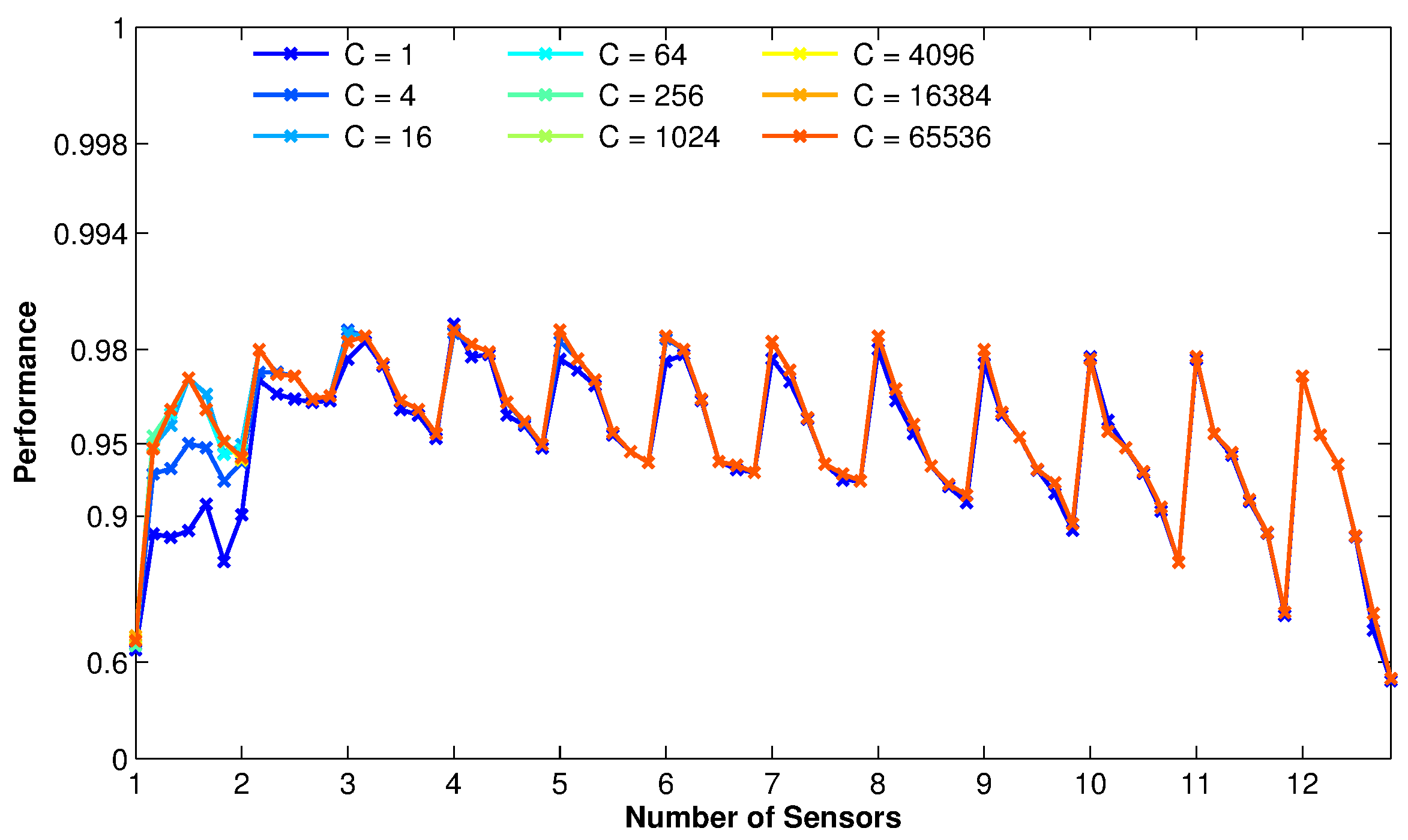

Supplement: Figure S9 — Average classification performance for all the test sets using radial SVM with different cost values and for the kernel function. The value used here is the inverse of the average of 0.5 quantile of all the pairwise distance of the data. The x-axis shows the sensor size constraints used in the feature selection, each tick mark represents the time point size constraint of 1, the performance values as marked out on the plots between two x-axis tick marks represents time point size constraints . The y-axis shows the performance in a highly non-linear, logarithmic scale. (TIF) [file pone.0089840.s009.tif]

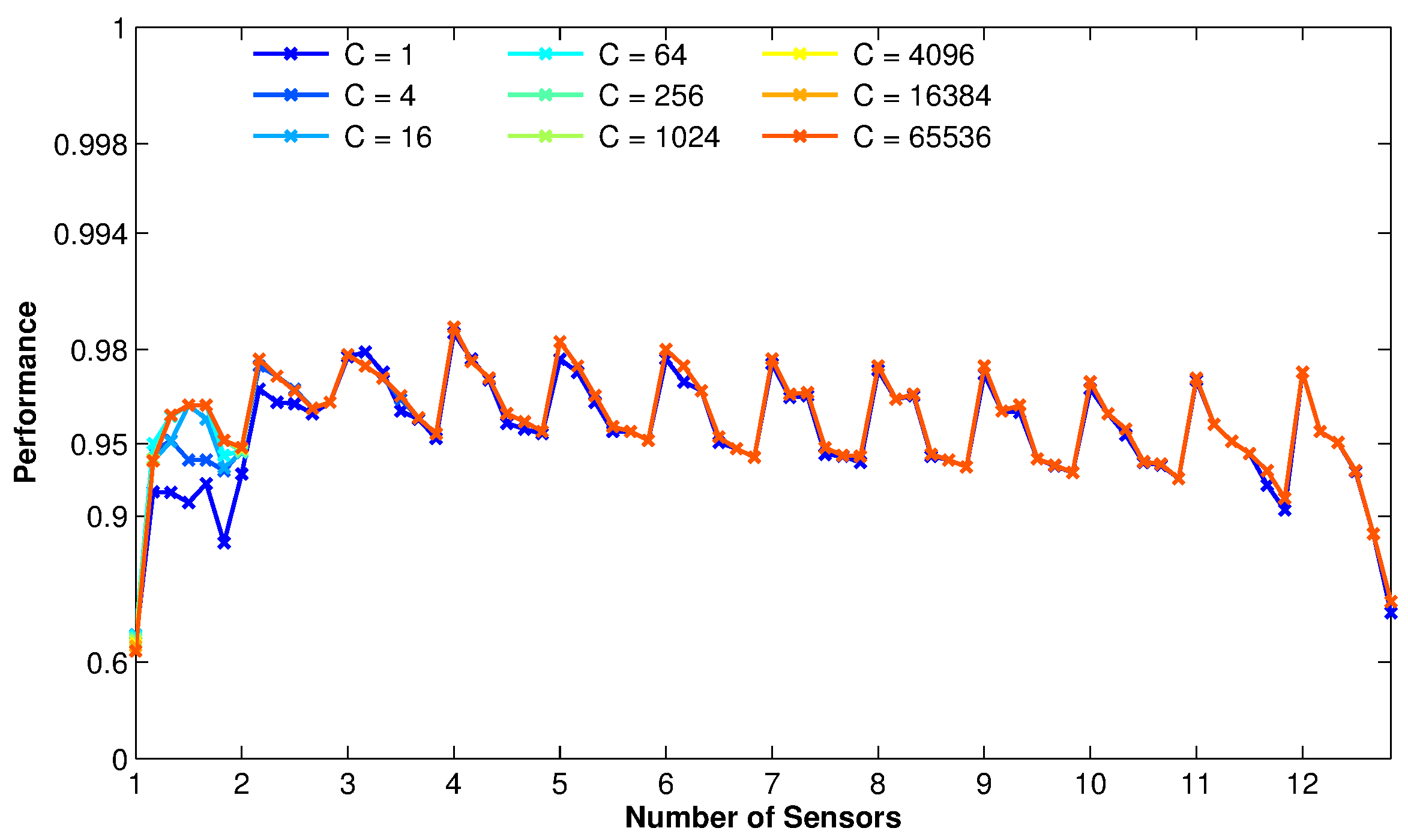

Supplement: Figure S10 — Average classification performance for all the test sets using radial SVM with different cost values and , where is the 0.5 quantile of the pairwise distance of the given feature set size, for the kernel function. The x-axis shows the sensor size constraints used in the feature selection, each tick mark represents the time point size constraint of 1, the performance values as marked out on the plots between two x-axis tick marks represents time point size constraints . The y-axis shows the performance in a highly non-linear, logarithmic scale. (TIF) [file pone.0089840.s010.tif]

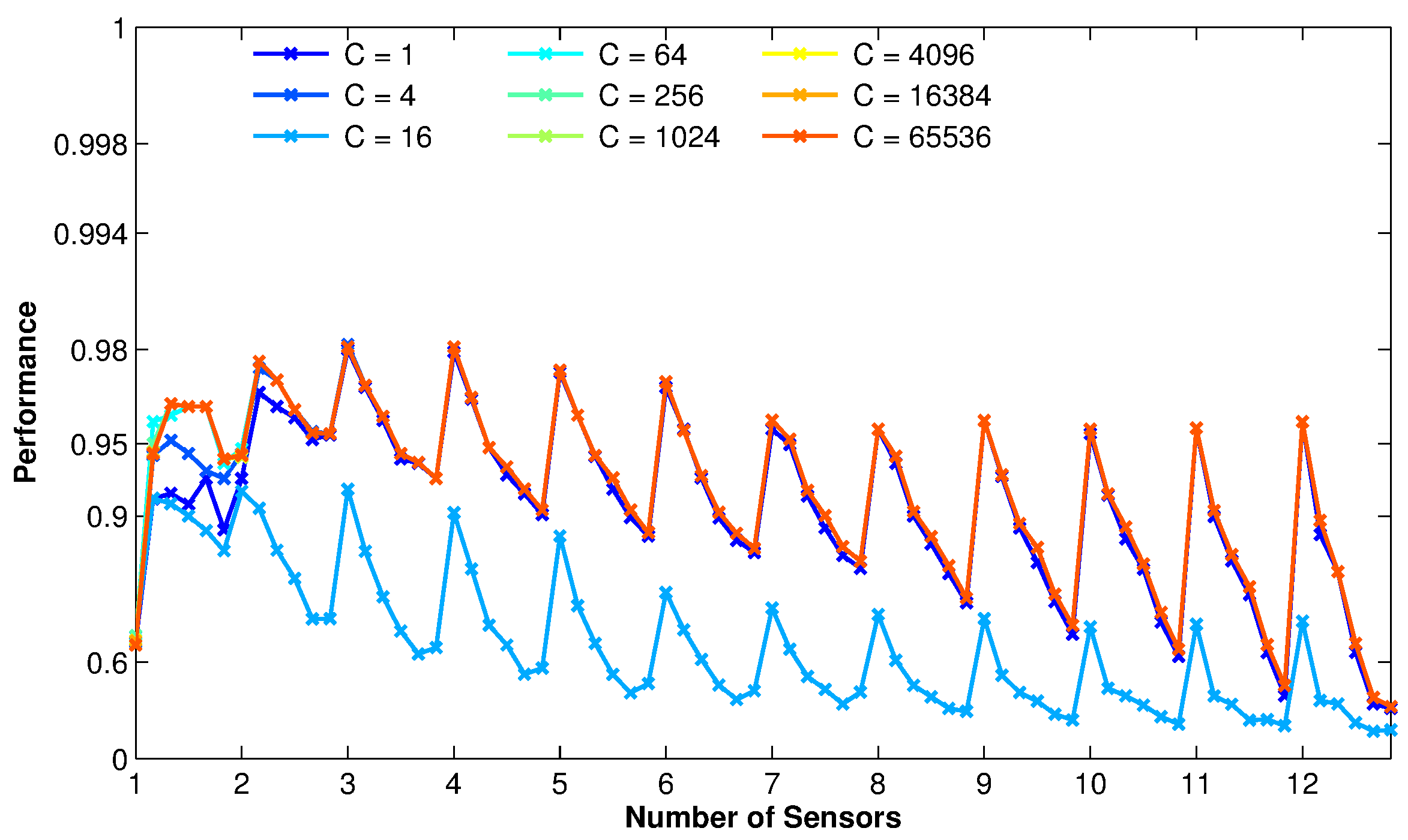

Supplement: Figure S11 — Average classification performance for all the test sets using radial SVM with different cost values and for the kernel function. The value used here is the inverse of the average of 0.1 quantile of all the pairwise distance of the data. The x-axis shows the sensor size constraints used in the feature selection, each tick mark represents the time point size constraint of 1, the performance values as marked out on the plots between two x-axis tick marks represents time point size constraints . The y-axis shows the performance in a highly non-linear, logarithmic scale. (TIF) [file pone.0089840.s011.tif]

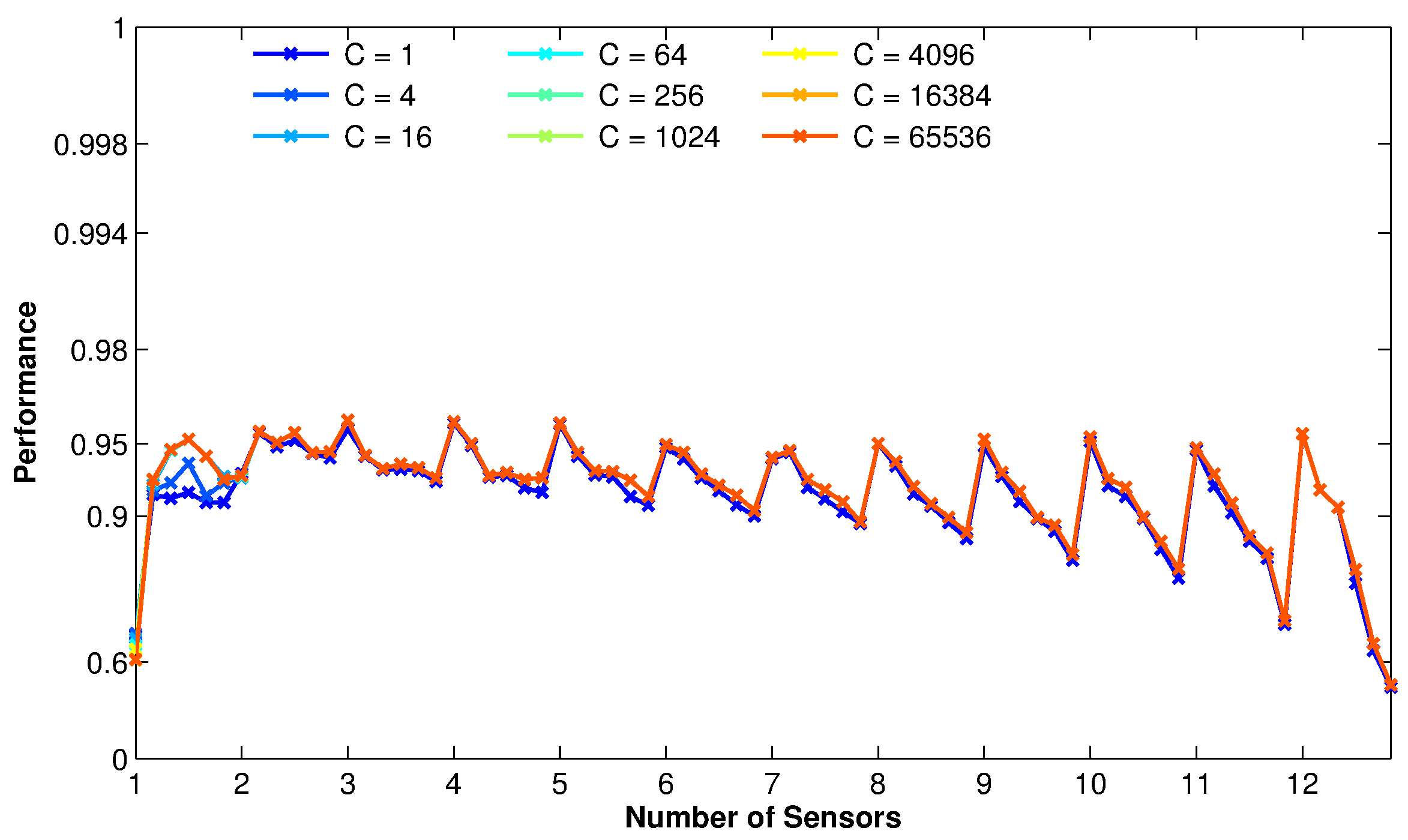

Supplement: Figure S12 — Average classification performance for all the test sets using radial SVM with different cost values and , where is the 0.1 quantile of the pairwise distance of the given feature set size, for the kernel function. The x-axis shows the sensor size constraints used in the feature selection, each tick mark represents the time point size constraint of 1, the performance values as marked out on the plots between two x-axis tick marks represents time point size constraints . The y-axis shows the performance in a highly non-linear, logarithmic scale. (TIF) [file pone.0089840.s012.tif]

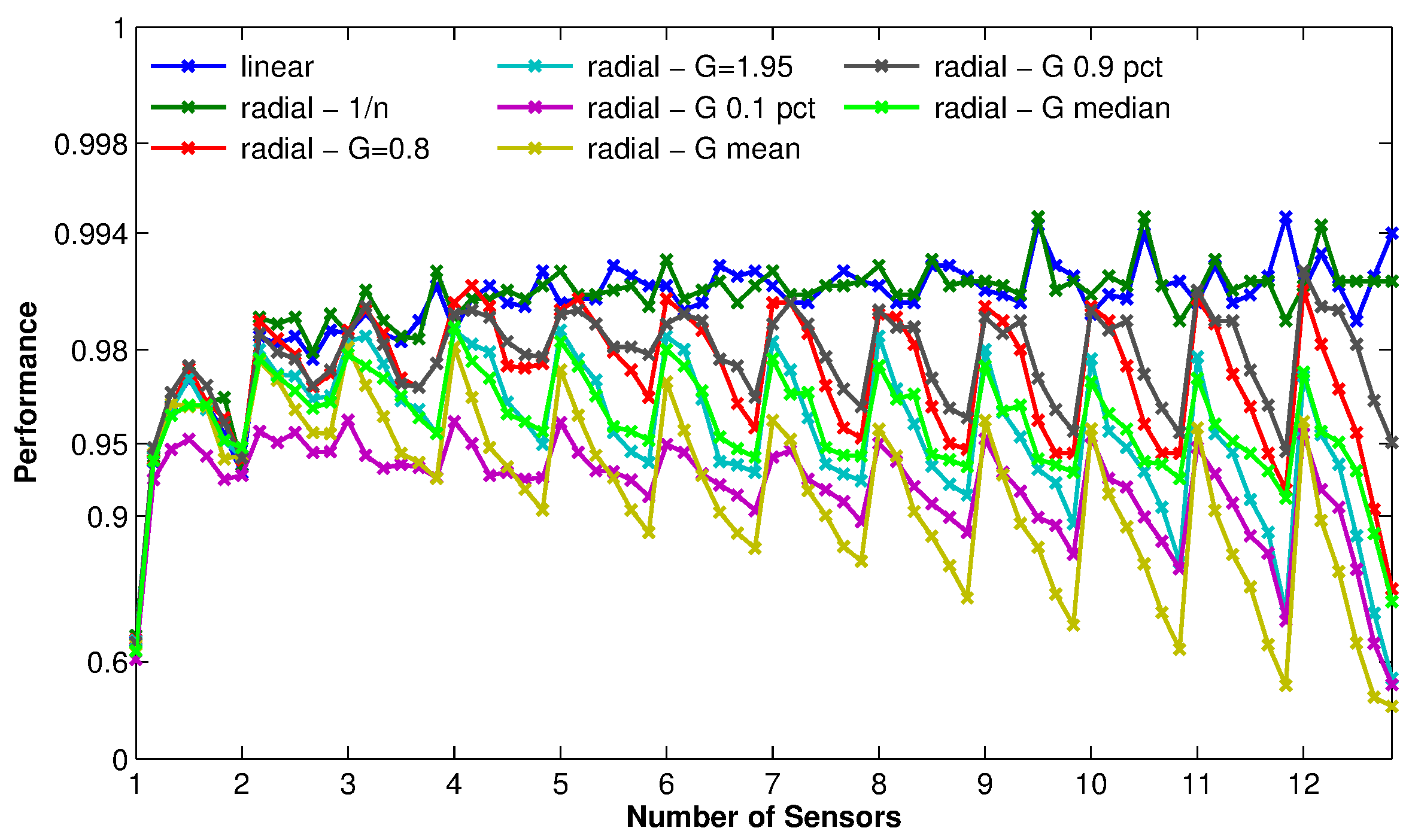

Supplement: Figure S13 — Comparison of average classification performance for all the test sets using linear SVM and radial SVM with different values for the kernel function. The cost value is set at , which is the value with the best performance for each individual kernel function. The x-axis shows the sensor size constraints used in the feature selection, each tick mark represents the time point size constraint of 1, the performance values as marked out on the plots between two x-axis tick marks represents time point size constraints . The y-axis shows the performance in a highly non-linear, logarithmic scale. (TIF) [file pone.0089840.s013.tif]
