# Supplementary material for: Feature Selection for Chemical Sensor Arrays Using Mutual Information
Source: PLoS One. 2014 Mar 4;9(3):e89840. doi: 10.1371/journal.pone.0089840 (PMC3942325; doi:10.1371/journal.pone.0089840)
Supplement: Table S1 — Overview of the sensors used in the electronic nose. A short description of the sensors is given here. CTO - Chromium-titanium oxide, SnO2 - Tin Oxide. (PDF) [file pone.0089840.s014.pdf]

Table S1. Overview of the sensors used in the electronic nose.

#	Sensor name	Description
1	CTO	CTO sensor without coating, general VOC sensor.
2	CTO-HZSM-5	CTO sensor with H-ZSM-5 zeolite overlayer, pore size 5.1 - 5.5 Å.
3	CTO+NaZSM-5	CTO sensor with Na-ZSM-5 zeolite overlayer, pore size 5.1 - 5.5 Å.
4	CTO+HLTA	CTO sensor with H-LTA zeolite overlayer, pore size 3.5 Å.
5	CTO+MCM-41	CTO sensor with MCM-41 overlayer, pore size 30 - 100 Å.
6	CTO+HZSM-22	CTO sensor with H-ZSM-22 zeolite overlayer, pore size 4.6 - 5.7 Å.
7	T30/1	SnO_2 sensor for detecting solvents.
8	P10/1	SnO_2 sensor for detecting hydrocarbons and methane.
9	P10/2	SnO_2 sensor for detecting methane, propane and aliphaic non polar molecules.
10	P40/1	SnO_2 sensor for detecting chlorinated and fluorinated compounds.
11	T70/2	SnO_2 sensor for detecting alcohol vapours and aromatic compounds.
12	PA/2	SnO_2 sensor for detecting low concentration of hydrogen, ammonia, amines.

A short description of the sensors is also given here. CTO - Chromium-titanium oxide, SnO_2 - Tin Oxide.
