# Supplementary material for: Feature Selection for Chemical Sensor Arrays Using Mutual Information
Source: PLoS One. 2014 Mar 4;9(3):e89840. doi: 10.1371/journal.pone.0089840 (PMC3942325; doi:10.1371/journal.pone.0089840)
Supplement: Table S2 — Chemical compounds analysed. The chemical are shown here under their group names and corresponding concentration. (PDF) [file pone.0089840.s015.pdf]

Table S2. Chemical compounds analysed.

Alcohols ($1.22 \times 10^5 M$)	Aldehydes (8.03×10^5 M)	Esters (3.70×10^5 M)	Ketones (3.79×10^5 M)
1-Pentanol	Acetaldehyde	Ethylhexanoate	Acetone
1-Hexanol	Butanal	Ethylacetate	2-Butanone
Z2-Hexen-1-ol	Hexanal	Isopentylacetate	2-Pentanone
1-Octen-3-ol	E2-hexenal	Methylacetate	2-Heptanone
3-Methylbutanol	Furfural	Ethylbutyrate	2,3-Butanedione

The chemical are shown here under their group names and corresponding concentration.
